# Unveiling the Effects of Interchain Hydrogen Bonds on Solution Gelation and Mechanical Properties of Diarylfluorene-Based Semiconductor Polymers

**DOI:** 10.34133/2020/3405826

**Published:** 2020-09-30

**Authors:** Lubing Bai, Yamin Han, Chen Sun, Xiang An, Chuanxin Wei, Wei Liu, Man Xu, Lili Sun, Ning Sun, Mengna Yu, He Zhang, Qi Wei, Chunxiang Xu, Yingguo Yang, Tianshi Qin, Linghai Xie, Jinyi Lin, Wei Huang

**Affiliations:** ^1^Center for Supramolecular Optoelectronics (CSO), Key Laboratory of Flexible Electronics (KLOFE) and Institute of Advanced Materials (IAM), Nanjing Tech University (NanjingTech), 30 South Puzhu Road, Nanjing 211816, China; ^2^State Key Laboratory of Bioelectronics, School of Electronic Science and Medical Engineering, Southeast University, Nanjing 210096, China; ^3^Center for Molecular Systems and Organic Devices (CMSOD), Key Laboratory for Organic Electronics and Information Displays & Institute of Advanced Materials (IAM), Nanjing University of Posts & Telecommunications, 9 Wenyuan Road, Nanjing 210023, China; ^4^Frontiers Science Center for Flexible Electronics (FSCFE), Shaanxi Institute of Flexible Electronics (SIFE), Shaanxi Institute of Biomedical Materials and Engineering (SIBME), Northwestern Polytechnical University (NPU), 127 West Youyi Road, Xi'an 710072, China; ^5^Shanghai Synchrotron Radiation Facility (SSRF), Zhangjiang Lab, Shanghai Advanced Research Institute, Chinese Academy of Sciences, 239 Zhangheng Road, Shanghai 201204, China

## Abstract

The intrinsically rigid and limited strain of most conjugated polymers has encouraged us to optimize the extensible properties of conjugated polymers. Herein, learning from the hydrogen bonds in glucose, which were facilitated to the toughness enhancement of cellulose, we introduced interchain hydrogen bonds to polydiarylfluorene by amide-containing side chains. Through tuning the copolymerization ratio, we systematically investigated their influence on the hierarchical condensed structures, rheology behavior, tensile performances, and optoelectronic properties of conjugated polymers. Compared to the reference copolymers with a low ratio of amide units, copolymers with 30% and 40% amide units present a feature of the shear-thinning process that resembled the non-Newtonian fluid, which was enabled by the interchain dynamic hydrogen bonds. Besides, we developed a practical and universal method for measuring the intrinsic mechanical properties of conjugated polymers. We demonstrated the significant impact of hydrogen bonds in solution gelation, material crystallization, and thin film stretchability. Impressively, the breaking elongation for P4 was even up to ~30%, which confirmed the partially enhanced film ductility and toughness due to the increased amide groups. Furthermore, polymer light-emitting devices (PLEDs) based on these copolymers presented comparable performances and stable electroluminescence (EL). Thin films of these copolymers also exhibited random laser emission with the threshold as low as 0.52 *μ*J/cm^2^, suggesting the wide prospective application in the field of flexible optoelectronic devices.

## 1. Introduction

Conjugated polymers (CPs) with intrinsic elasticity property are desirable for deformable optoelectronic devices, such as organic photovoltaics (OPV) [1, 2], polymer light-emitting diodes (PLEDs) [3], and wearable sensors [4, 5]. So far, extensive efforts have been made to improve their optoelectronic performances instead of CPs mechanical performance metrics. For most CPs, plentiful *π* − *π* stacking interactions among the conjugated backbones always induced the polymer chains stay in glassy states, thus resulted in material crystallization. It is meaningful to develop viscoelastic and intrinsic stretchable CPs, of which their polymer chains are incompact entanglement. Meanwhile, predicting and directly measuring the intrinsic mechanical properties of CPs remain a daunting task [6]. One hurdle is that the laboratory-scale materials cannot be processed into freestanding thin films for the conventional pull test; another hurdle is the limited toolbox of techniques for precisely assessing their intrinsic mechanical properties [7]. Metrics of the mechanical properties, including the elastic modulus and hardness, are helpful for evaluating their application in flexible and stretchable optoelectronic devices. The inherent *π*-conjugated structure and limited strain of most CPs prompt us to optimize their elastic properties with a minimal compromise of optoelectronic performances.

In general, it is challenging for CPs to simultaneously possess superior optoelectronic performance and outstanding thin film ductility. Superior thermal stability of thin film morphology is prerequisite to achieve excellent optoelectronic performances, especially for the PLEDs, of which the active layer always suffer from the Joule self-heating effect while working [8]. The essence of polymer viscoelastic and stretchability are intrachain conformational transition, interchain entanglement, and disentanglement, which generally require low activation energy of chain segmental motion [9]. For CPs expected to endure tensile modes of deformation, ~20% strain can sufficiently fulfil the requirement of flexible and wearable devices in most cases. Compared to the gum-like CPs at room temperature, viscoelastic CPs (similar to drawable plastic) with low tensile modulus and toughness are the most potential candidates [10, 11]. So far, one common approach is incorporating nonconjugated flexible linker into the backbone, such as the linear alkyl chain with varying length and the spacer that can form dynamic reversible cross-links (similar to the interchain entanglement point) [12–15]. Being weaker than the covalent bonds, dynamic noncovalent interactions, such as *π* − *π* stacking interactions [16–18], hydrogen bonds [19–21], and metal-ligand interactions [22–24], endow materials with abundant self-assembly and stimuli-responsive abilities. Therefore, developing stretchable CPs demands a much deeper understanding about how the intermolecular interactions affect the mechanical properties from either microscale or macroscale.

**Figure 1:**
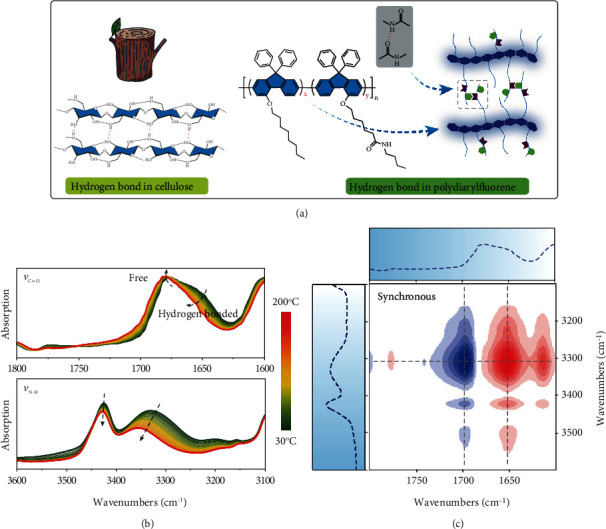
Schematic illustration of amide-functionalized polydiaryfluorene and characterization of hydrogen bonds. (a) Hydrogen bonds among the neighbouring chains. Chemical structure of P (ODPF-*co*-ADPF) and the schematic illustration on the intermolecular hydrogen bonds. (b) Variable temperature FT-IR spectra of P4 ranged from 30°C to 200°C: the 1600-1700 cm^−1^ region due to C=O vibrations, 3100-3600 cm^−1^ region due to N-H vibrations. (c) Synchronous 2D-FTIR correlation spectra of P4 as a function of temperature, calculated with software of 2Dshige according to the spectra data of C=O (top) and N-H (right).

In addition, CPs with substantially enhanced carrier mobility, and microscale crystallization has also been enabled through reasonable side-chain modifications, such as branched alkyl chains [25, 26], urea containing alkyl chains [27], and triethylene glycol chains [28]. These functionalized side chains have a crucial consequence on the intermolecular interactions, and hence on chains packing structure as well as thin film morphology, and possibly on the toughness and strength of CPs films. In this work, inspired by the strong cellulose nanofiber in the natural [29], whose mechanical performance has been greatly improved by hydrogen bonds among the aligned glucose chains (Figure 1(a)), we introduced varied ratios of amide-containing alkyl side chains (10%, 20%, 30%, and 40%) into polydiarylfluorene through random copolymerization, in order to investigate the influence of interchain hydrogen bonds to their condensed state properties and thin film mechanical performances. Nuclear magnetic resonance (NMR) and variable temperature Fourier transform infrared (FT-IR) spectroscopy were taken to ascertain the formation of interchain hydrogen bonds. Compared to the investigation of DPP-based polymers from Simon et al. [30], we further studied the influence of amide-containing proportion on solution gelation, which is also significant for the solution-processed technologies in optoelectronics. Most importantly, we developed a practical method for measuring the intrinsic mechanical properties of thin films taking advantage of the conventional tensile strain instrument (dynamic mechanical analyzer, DMA), which is assisted by a hollow substrate (PMMA). The freestanding thin film (~800 nm) can be transferred easily to the tensile strain fixture. We demonstrated that, upon the increased ratio of amide-containing side chains, the interchain interactions were more and more intense. When the ratio reached 30% and 40%, their semidilute toluene solution showed gelation while their viscosity exhibited a feature of the shear-thinning process, which resembled the non-Newtonian fluid. Mechanical properties, including Young's modulus and breaking elongation, also enhanced to some extent upon the amide proportion increasing. What is more, combining the analysis of optical spectra and the performance of PLEDs devices, we systematically investigated the impact of increased hydrogen bonds on the optoelectronic properties. Finally, all of the investigated copolymers exhibited impressive random laser emission with remarkable thresholds at a range from 0.52 *μ*J/cm^2^ to 1.38 *μ*J/cm^2^, suggesting the wide prospective application in the field of organic lasers.

## 2. Results

### 2.1. Preparation and Structural Characterization

All investigated copolymers with different amide ratios (10%, 20%, 30%, and 40%, named as P1, P2, P3, and P4) at the side chains were prepared through the Yamamoto random copolymerization by varying the molar ratio of monomer A and B from 9 : 1 and 4 : 1 to 7 : 3 and 3 : 2 (see detailed procedures in Scheme S1). The detailed information for preparing monomer B (Scheme S1) and characterization of molecular structures (Figure S1) were also attached. We verified the chemical structures of P1 to P4 by ^1^H-NMR and precisely calculated the copolymerization ratio of monomer B according to the integrated area of characteristic peaks, including the peaks of protons near the ether linkage (4.22 ppm) and the carbonyl of amide group (3.2-3.3 ppm) (Figure S2). The calculated ratios for P1-P4 were 10%, 20%, 29%, and 40%, respectively, which were consistent with the theoretical ratios calculated by feed ratios (Table S1). The broad signal of the N-H group was detected to be 5.42 ppm. After adding few drops of protic solvent (methanol-*d*^4^), the signal disappeared due to proton exchange, which further confirmed the existence of amide groups (Figure S3). The number-average molecular weight (*M*_n_) and polydispersity index (PDI) of these copolymers were measured with gel permeation chromatography (GPC) using dimethylformamide (DMF) as the eluent (Table S2, Figure S4). Thermogravimetric (TG) analysis demonstrated that the decomposition temperatures (*T*_d_) of P1-P4 were >400°C (Figure S5(a)), indicating their superior thermal stability. In addition, differential scanning calorimetry (DSC) was performed to conduct a comparative evaluation of P1-P4 thermodynamic property, as shown in Figure S5(b). Compared to the heat flow curves of polydiarylfluorene without amide groups at the side chains (named as PODPF) [31–33], the amide-functionalized CPs exhibited decreased and less obvious melting temperatures (*T*_m_). Meanwhile, P1 and P2 presented a more distinct glass transition process either in the heating or cooling process with the glass transition temperature (*T*_g_) of 170°C, lower than the *T*_g_ (192°C) of PODPF. P3 and P4 exhibited a distinct melting crystallization process (*T*_mc_) at the temperature of 213°C, lower than that of PODPF (277°C), while this process was absent in P1 and P2. These results indicate that incorporating amide groups was facilitated to the material crystallization.

To further confirm the formation of hydrogen bonds among the intermolecular side chains, we performed variable temperature FT-IR spectroscopy and taken P4 as the representative sample (Figure S6).  Figure 1(b) shows the magnified spectra of 1640~1700 cm^−1^ and 3300~3500 cm^−1^, which were, respectively, assigned to the stretching mode of carbonyl groups (C=O) and N-H groups (Figure S7). Upon increasing the temperature from 30°C to 200°C, the FT-IR intensity and wavenumbers of characteristic peaks changed gradually as a result of interchain hydrogen bonds breaking. The intensity of shoulder peak at 1650 cm^−1^, corresponding to the hydrogen-bonded carbonyl groups, decreased substantially upon the temperature increase. The peak intensity at 1680 cm^−1^ slightly enhanced and shifted to high wavenumbers, which was ascribed to the increased amount of free carbonyl groups (Figure 1(b), top). Correspondingly, the peak intensity of N-H groups at 3330 cm^−1^ decreased and shifted to 3355 cm^−1^ due to the break of hydrogen bonds (Figure 1(b), bottom). Then, we conducted three heating and cooling cycles for the varied temperature FT-IR test, and these changes were completely reversible (Figure S8), which strongly demonstrated the formation of intermolecular hydrogen bonds. In order to get further insight about the correlation of these peaks variation upon increased temperature, we calculated and depicted the synchronous 2D-FTIR correlation spectroscopy according to the spectra data of C=O and N-H vibrations. As shown in Figure 1(c), we observed that the peak intensity at 3330 cm^−1^ exhibited a strong negative correlation to the peak intensity at 1680 cm^−1^, while a strong positive correlation to the peak intensity at 1650 cm^−1^. This observation manifested that the spectra in this region were strong correlation, which further confirmed the formation of supramolecular hydrogen bonds among the C=O and N-H groups in polymer side chains.

### 2.2. Hierarchical Condensed Structures of CPs

**Figure 2:**
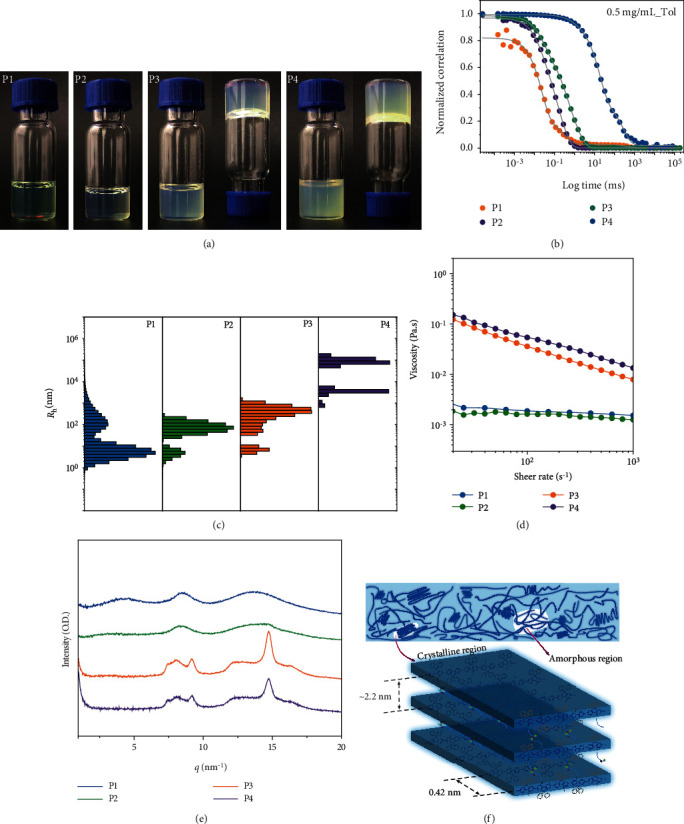
Hydrogen bond effects on solution gelation, viscosity, and crystallization. (a) The semidilute toluene solution of P1-P4 (10 mg/mL) before and after aging at room temperature for 10~20 min. (b) Normalized intensity correlation function (*g*^2^(*t*) − 1) of P1-P4 in dilute toluene solution (0.5 mg/mL), measured at the scattering angle of 90^o^. (c) The hydrodynamic radius (*R*_h_) distributions of P1-P4 in dilute solutions calculated from dynamic light scattering (DLS) measurements. (d) Rheological measurement for P1-P4 in 10 mg/mL toluene solution: their respective shear sweep viscosity as a function of shear rate. (e) Wide-angle X-ray scattering (WAXS) data for P1-P4 drop-coated films. (f) Crystallization dominated polymer films and schematic representation of the edge-on molecular packing in the crystalline region.

Optimizing the molecular packing and film morphology through side-chain modification was a facile and concise strategy. Previous reports have proven that side chains, especially those containing crosslink moieties (amide or urea groups, etc.), have drastically influence on the interchain interactions of conjugated polymers [27, 34]. Firstly, we investigated the effect of interchain hydrogen bonds on the CPs solution properties by carrying out the aging measurements for their semidilute toluene solution (10 mg/mL). As shown in Figure 2(a), all the copolymers investigated in this work dissolved well in toluene after stirring and heating. Impressively, the solution of P3 and P4 formed semitransparent gels upon cooling down to room temperature and aging for 10~20 min, while P1 and P2 maintained homogeneous solution. In order to precisely characterize this transformation, we mixed P2 and P3 solution with various ratios, as summarized in Table S4, and the solution gelation emerged in the amide-containing ratio of 27.5%, as shown in Figure S9. We attributed this solution gelation to the increased amount of interchain hydrogen bonds formed among amide-containing side chains: the higher amide side chains proportion, the more cross-linked hydrogen bonds. Interestingly, the P3 and P4 gels could turn to fluidic state upon shaking and return to the gel after the shaking stopped and aging for several minutes. The reversible transformation between fluid and gel was ascribed to the disruption and formation of hydrogen bonds, suggesting that the enhancement of interchain dynamic interactions have significant impact on the solution property of CPs. However, when dissolved in chlorobenzene, none of their solution could form supramolecular gels no matter how long the solution aging, because the halogen bond (C-Cl) of chlorobenzene destroyed the formation of interchain hydrogen bonds (Figure S10). It is well known that hydrogen bond is a versatile and universal gel-forming supramolecular interaction, which has been fully exploited in the field of hydrogel [35]. The observations above confirmed that, through reasonable design, reversible gelation induced by dynamic noncovalent bond is equally applicable to CPs solution, and the controllability of the solution properties can be imparted through adjusting the proportion of hydrogen bonds. In order to understand the internal mechanism of gelation and quantitatively evaluate the relationship between the aggregation behavior of CPs in solution and the proportion of hydrogen bonds, we carried out dynamic light scattering (DLS) analysing.  Figure 2(b) displays the typical normalized correlation function (*g*^2^(*t*) − 1) of P1-P4 measured at a concentration of 0.5 mg/mL. We observed that the correlation function shifted to longer lag time with the ratio of amide side chains increased from 10% to 40%, reflecting an increased hydrodynamic radius (*R*_h_) as a result of the increased amount of intermolecular hydrogen bonds.  Figure 2(c) presents the *R*_h_ distribution fitted from the correlation curves, that all the solution displayed two relaxation modes (i.e., fast mode and slow mode). The fast mode with the average *R*_h_ ranged from 1 nm to 30 nm was attributed to the translational diffusion of single polymer chains, and the slow mode appeared at above 100 nm was considered to be the cross-linked polymer aggregates due to the intermolecular interactions [36]. Obviously, the fast mode in P1 is the main part, indicating that the polymer chains in toluene solution mainly existed in the form of single chains. When the proportion of amide side chains increased to 20% and 30%, the slow mode turned to be the main part, reflecting that the polymer chains mainly exist in the form of cross-linked aggregates due to the increased amount of interchain hydrogen bonds. The correlation function changed markedly when the ratio increased to 40%, and an additional relaxation mode appeared with an average *R*_h_ of about 1000 nm, indicating the formation of larger and more compact aggregates. Combined with observations in the aging measurements, we were more convinced that cross-linked hydrogen bonds among intermolecular side chains induced the gelation of P3 and P4. Furthermore, we investigated the viscosity of their toluene solution (10 mg/mL) by rotational rheometry at various shear rates (Figure 2(d)). The viscosity of P1 and P2 was at about 2 × 10^−3^ Pa · s, which showed negligible change during the shear sweep process. In respect to P3 and P4, they exhibited a shear-thinning process, and their viscosity reduced from 0.15 Pa · s to 8 × 10^−3^ Pa · s. This is a general feature of the non-Newtonian fluids, which resulted from the break of interchain hydrogen bonds under external force. These observations are coincident with the shaking experiment mentioned above. The film morphologies of P3 and P4 spin-coated from the toluene solution were rougher than that from chlorobenzene, and no matter from which solution, the film roughness increased upon the increased amount of amide groups (Figure S11). Therefore, we concluded that interchain hydrogen bonds would largely enhance the intermolecular interactions of CPs, which would drastically influence their solution state and solution-processed film morphology.

The mechanical properties are determined not only by the individual molecules structure but also by their molecules' packing structure in the solid state [37]. In order to examine whether the intermolecular hydrogen bond facilitated a long-range order molecular packing structure, wide-angle X-ray scattering (WAXS) and grazing-incidence X-ray diffraction (GIXRD) were carried out to study the drop-coated films of P1-P4. As shown in Figure 2(e), P3 and P4 exhibited more pronounced Bragg diffraction peaks upon the increased amount of hydrogen bonds, indicating that P3 and P4 were more easily crystallized than P1 and P2, consistent with the analyzed result in Figure S5. The increased crystallinity observed in these diarylfluorene-based polymers was in contrast to the reported results observed in the DPP-based polymers, which was one kind of easily crystalline conjugated polymers. This observation was in good agreement with the diffraction patterns observed from the GIXRD measurement, which showed well-defined out-of-plane peaks for P3 and P4 (Figure S12). From the scattering vector (*q*) at 14.8 nm^−1^, the *π* − *π* stacking distance of 0.42 nm was determined. The peak at *q* of 9.2 nm^−1^ was calculated to be 0.68 nm, closed to the length of single fluorene molecular (0.887 nm) [38]. The lamellar packing distance (interchain distance) was determined to be approximately 2.2 nm from the diffraction peak at *q*_*z*_ of 2.8 nm^−1^ measured by GIXRD. This result coincided with the interchain distance of 20.5 Å calculated from the peak at 2*θ* of 4.3^o^, measured by X-ray diffraction (XRD, Figure S13). The derived arrangement of this long-range-order packing structure is schematically presented in Figure 2(f), illustrating an edge-on packing orientation of the polymer backbone with cross-linked hydrogen bond among the intermolecular side chains. All of these results above revealed that upon the increased proportion of amide side chains, P3 and P4 films exhibited more well-organized stacking structure with lamellar oriented normal to the substrate, suggesting that introducing interchain hydrogen bonds was a critical molecular design strategy for realizing long-range-order polymer packing structure [39]. Predictably, the distribution of microscale crystalline structure in spin-coated films may reasonably enhance their toughness and strength.

### 2.3. Intrinsically Mechanical Properties of These Conjugated Polymers

**Figure 3:**
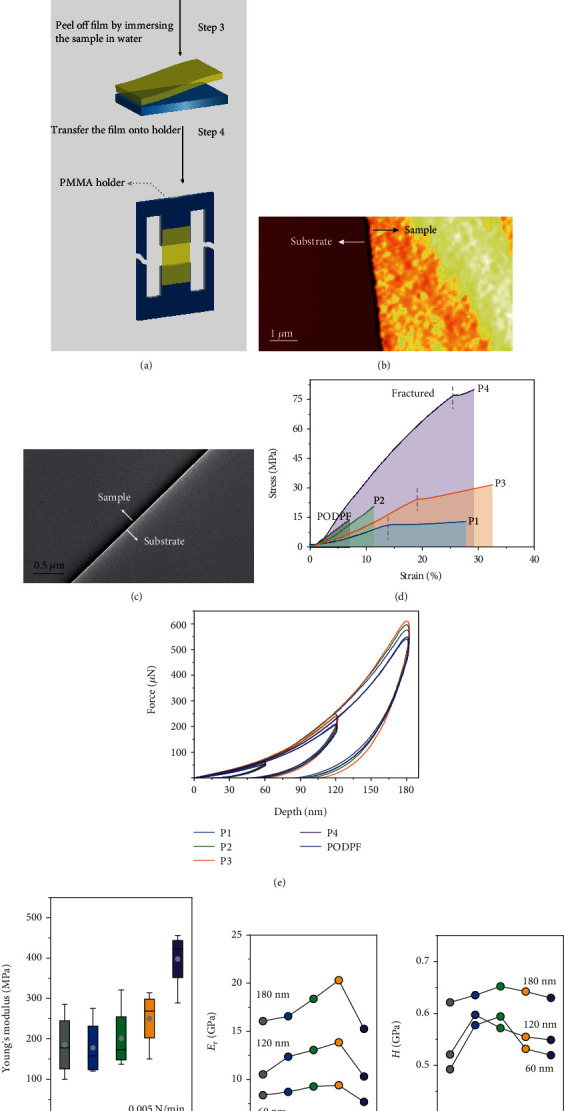
Sample preparation for the stress-strain test and mechanical properties. (a) Schematic of sample preparation for conventional tensile test. (b) AFM and (c) TEM image of the film edge. (d) Comparison of tensile stress-strain curves for P1-P4 films. (e) Force-depth curves recorded by nanoindentation test. (f) Young's modulus achieved by the conventional tensile test. (g) Elastic modulus and (h) hardness achieved by nanoindentation with the loading depth of 60, 120, and 180 nm.

Although the performance of flexible optoelectronic devices based on CPs has been greatly developed, there has been few of exalting improvement about the intrinsically mechanical properties of materials, which is mainly hindered by the feasible stretching method. The currently developed strategies, such as the water-supported direct tensile test (film-on-water, FOW) or elastomer-supported buckling-based method (film-on-elastomer, FOE), still remain some drawbacks need to be overcome, including how to eliminate the deviation caused by the surface tension of water (73 mN m^−1^) [9, 40]. Herein, we introduced a feasible method taking full advantage of the conventional tensile instrument, for conveniently evaluating mechanical metrics of CPs. The key step in sample preparation is to make transferable film, as shown in step 3 of Figure 3(a). After immersing the spin-coated film in water, the film detached from the quartz substrate and suspended on the water surface. Then, we could readily pick up this film assisted by the specially designed hollow holder and transfer it onto the tensile test fixture (Figure S14, detailed description in methods). The preparation for freestanding membrane is a wholly solution-based process, and suitable for laboratory-scale materials. The images of the floated film taken by AFM (Figure 3(b)) and transmission electron microscope (TEM, Figure 3(c)) revealed that the film spread out under the surface tension of water, and there is no fracture on edge.  Figure 3(d) is the typical stress-strain curves obtained by this strategy with a loading rate of 0.005 N/min, while Figure 3(f) shows Young's modulus of P1-P4 and the preferred sample (PODPF). We have performed this tensile test for several samples of each CPs, in order to minimize the combined effect of inhomogeneity in film thickness as well as aggregate domains in film morphology. Obviously, we observed that all the CPs were brittle fracture, and the curves after the fracture point in P1, P3, and P4 resulted from film tearing under stress instead of plastic yielding. The elongation at fracture for P3 and P4 was 19% and 25%, larger than other samples, which were 13% for P1, 11% for P2, and 7% for PODPF. In respect to Young's modulus of samples that the amide ratios were below 20%, no clear tendency was observed, and their mean values were approximately 200 MPa for P1, P2, and PODPF. When the amide ratio reached 30%, the mean elastic modulus increased slightly to 255 MPa, and when the amide ratio reached 40%, it further increased significantly to 400 MPa. Compared to P3HT tensile modulus (*M*_n_ = 15 kDa) obtained through FOW and FOE methods [9, 41–43], as summarized in Table S3, the modulus of CPs measured through our tensile strategy were on the same order of magnitude and a slightly higher than the reported values. We attributed this mainly to the discrepancy of molecular structure rigidity of fluorene and thiophene, and beyond this, the sample thickness and the strain rate applied on the films also account for this discrepancy. Furthermore, we carried out nanoindentation test to evaluate their mechanical properties, which has been widely applied in the research of crystal and metal, but rarely for CPs [44].  Figure 3(e) shows the load-displacement data at different load depths: 60, 120, and 180 nm. The elastic modulus (*E*_r_) (Figure 3(g)) and hardness (*H*) (Figure 3(h)) were, respectively, calculated according to formulas (1) and (2), as shown in supplied methods. We observed that the *E*_r_ values attained by nanoindentation were tens of times larger than Young's modulus measured by the tensile test, which was slightly increased from 16 GPa of PODPF to 20 GPa of P3 and decreased to 15 GPa of P4 under the loading depth of 180 nm. These results were in consistent with the nanoindentation results of polyfluorene derivatives reported by Zeng et al. [45], and the slight discrepancy may result from the indenter shape. We speculate that the enhancement was resulted from the chain entanglement induced by hydrogen bond cross-linking. As for the abrupt decrease, we infer it may be the result of the rough morphology and the fluffy feature induced by the gelation of P4. Despite no identified tendency in the *H* values, these results were closer to the tensile modulus obtained from the tensile test. On the other hand, these values showed to gradually increase as the loading depth increase, which ascribed to the substrate effect under larger loading depth. Therefore, nanoindentation is not the preferred method for testing the thin film of conjugated polymer. We also characterized their stretchability through the FOE method by using a simple biaxial tension device (Figure S15). We observed that PODPF and P1 emerged cracks under 5% strain, and all the polymers showed lots of cracks under 10% strain. But for P3 and P4, their films were still consecutive, and until the strain reached to 15%, all the films were cracked (Figure S16). However, because we cannot manipulate this loading rate precisely, we did not obtain the accurate Young's modulus. Taking these observations into consideration, we were more convinced about the effectiveness and practicability of this tensile strategy. We believed that, as stress dissipation parts, the reversible hydrogen bonds have a remarkable contribution for enhancing the stretchability of semiconductor polymers.

### 2.4. Photophysical Properties and PLED Devices

**Figure 4:**
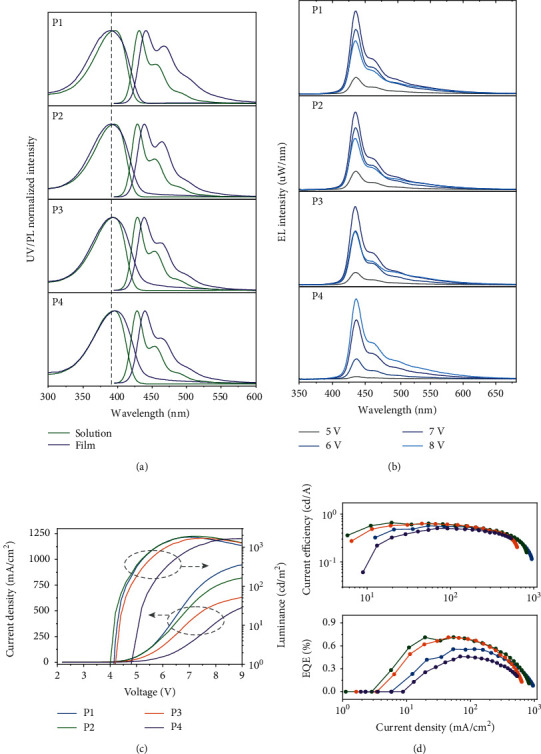
Photophysical properties and PLEDs performances. (a) Normalized UV-*vis* and PL spectra of P1-P4 in dilute toluene solution (green line, 10^−5^ M) and spin-coated films (violet line). (b) The electroluminescence (EL) spectra of devices based on P1-P4 upon the applied voltage increasing. (c) Current density-luminance-voltage (*J* − *L* − *V*) characteristics of PLED devices. (d) Current efficiency and external quantum efficiencies (EQE) versus current density curves of these devices.

In this section, we mainly focus on elucidating the photophysical properties of these amide-functionalized copolymers. The UV-*vis*, PL spectra, and photoluminescence quantum yield (PLQY) of every copolymers in film or solution were measured. As shown in Figures 4(a), the UV-*vis* and PL spectra for P1-P4 in toluene were almost identical, the peak absorption were at 395 nm, and the PL spectra had two pronounced peaks at 430 nm (0-0 transition) and 454 nm (0-1 transition), demonstrating the extended configuration of copolymer chains in dilute solution without being affected by the increased hydrogen bond proportion. However, for the absorption spectra of spin-coated films, a slight red shift was observed upon the increased amide-containing ratio: 389 nm for P1, 390 nm for P2, 392 nm for P3, and 394 nm for P4, which might stem from the film aggregates that resulted from the cross-linked gelation network. In addition, the tail regions in P3 and P4 were slightly larger than those in P1 and P2, mainly due to the light scattering resulting from the rough film morphology. In respect to the PL spectra of films, all the samples presented pronounced vibrational structure with the higher spectra intensity of *I*_0−0_ (439 nm) than that of *I*_0−1_ (465 nm), suggesting that there were little aggregates in films as well as small configurational relaxation in the excited state of P1-P4. The fluorescence lifetime (*τ*) in solution and films were determined by time-correlated single photon counting (TCSPC), which were approximately 0.3 ns in solution and 0.5 ns in films (Figure S17). The average PLQY values of P1-P4 films were 0.34 ± 0.05, 0.42 ± 0.05, 0.37 ± 0.05, and 0.38 ± 0.05, respectively. The highest occupied molecular orbital (HOMO) energy levels were determined by the cyclic voltammetry (CV), as shown in Figure S18. Furthermore, solution-processed PLEDs devices adopted P1-P4 as emitting layers were fabricated with the configuration of ITO/PEDOT: PSS (40 nm)/EML (30~40 nm)/TPBi (20 nm)/LiF (0.8 nm)/Al (100 nm) (see the detailed fabrication procedure in methods). The EL spectra of their PLEDs upon the increased applied voltage from 5 V to 8 V are shown in Figure 4(b). With the increased amide proportion of the side chains, the emission spectra showed negligible differences, and the peak wavelengths were at 436 nm for the investigated polymers, which were in consistent to the corresponding PL spectra of spin-coated films. Only the tail spectra of P3 and P4 were more obvious relative to P1 and P2 in high applied voltage, mainly owing to the larger morphology roughness that resulted from higher viscosity of the solution.  Figure 4(c) shows the current density-luminance-voltage (*J* − *L* − *V*) characteristics of the devices. The *J* − *V* curves for P1 and P2 exhibited less leakage current than P3 and P4 because of their homogeneous smooth surface topography. The turn-on voltage of devices based on P1-P3 was approximately 4.2 V, lower than 4.7 V of devices based on P4. The maximum luminance of these PLEDs were 1809 cd/m^2^ and 1913 cd/m^2^ for P1 and P2 at 7 V, slight higher than that of 1689 cd/m^2^ for P3 at 7.4 V, and 1658 cd/m^2^ for P4 at 9 V. We attributed this difference mainly to the much rough film morphology of P3 and P4, which resulted into the increased contacted resistance and reduced the formation of Ohmic contact, and further affected the recombination of injected holes and electrons in the emitting layer. Their current efficiency profiles were almost identical above the current density of 100 mA/cm^2^, indicating similar carrier behavior in emitting layers as shown in Figure 4(d). However, PLEDs based on P2 and P3 showed improved EQE, in contrast to the poor EQE of P1 and P4. The peak EQE for P2 and P3 is 0.7%, better than 0.6% of P1 and 0.5% of P4, which were comparable to the state-of-the-art performance of fluorene-based PLEDs. However, in regard to the problem of efficiency roll-off, no evident optimization was observed after introducing amide groups, suggesting we should afford more efforts for improving the PLEDs performance. From the results above, we conclude that the proportion of amide groups should be controlled within a certain range if we want to obtain superior device performance, preferably no more than 30%, with the aim of avoiding the negative effects resulted from intense interchain interactions.

### 2.5. Characterization of the Optical Gain Properties

**Figure 5:**
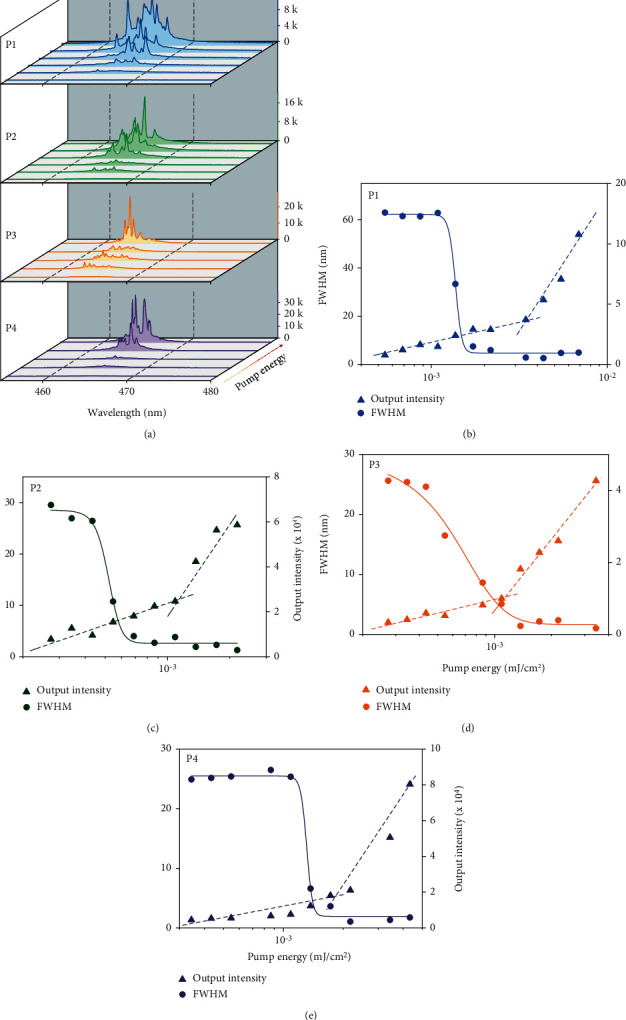
Properties of random laser emission. (a) The edge emission spectra of P1-P4 films as a function of increasing pump energy (*λ*_ex_ = 355 nm). (b–e) The full width at half maximum (FWHM) and output intensity versus input pump energy density for P1-P4, respectively.

Light-emitting conjugated polymers (LCPs) with superior optical gain properties can be exploited for the fabrication of distributed feedback (DFB) lasers. Intending to evaluate the feasibility of P1-P4 on the application of organic laser, we characterized the optical gain properties of thin films under femtosecond pulsed excitation (355 nm).  Figure 5(a) showed the edge-detected emission spectra of P1-P4 in thin film slab waveguides structures at different pump energy. For all investigated polymers, over a certain pumping energy, random laser emission was found and superimposed on the amplified spontaneous emission (ASE) band, and their spectra collapsed into multiple discrete emission peaks with the linewidth was about 1 nm. All of the peak wavelengths were about 465 nm, which range in the 0-1 vibrionic transition of their PL spectra, which were coincident with the general feature of four-level laser system. As shown in Figures 5(b)–5(e), the random laser thresholds (*E*_th_) were determined to be 1.38 *μ*J/cm^2^ for P1, 0.52 *μ*J/cm^2^ for P2, 0.70 *μ*J/cm^2^ for P3, and 1.32 *μ*J/cm^2^ for P4, with nonrelevance to the amide proportion, indicating the high optical gain coefficients and low waveguide scattering loss in thin films. The full width at half maximum (FWHM) of the emission spectra decreased drastically with the increased pump energy, from dozens of nanometers to 4.9 nm of P1, 1.8 nm of P2, 1.7 nm of P3, and 1.4 nm of P4. It is worth mentioning that the *E*_th_ reported here are considerably lower than the previously reported values of CPs-based gain medium. One of the possible explanation was that the amide-functionalized CPs possess excellent optical gain properties. On the other hand, we speculated that the random laser emission originates from the inside random microcavity, and the aggregates induced by interchain hydrogen bonds were actually responsible for the sharp random laser emission. According to the method reported in previous literatures [46, 47], the microcavity diameter of random laser emission was calculated by the Fourier transform (FT) of laser emission spectrum, which was approximately 30~60 *μ*m (Figure S19). Overall, we conclude that these hydrogen bond-functionalized polydiarylfluorenes exhibit great potential in the application of organic laser.

## 3. Discussion

In summary, we successfully prepared amide-functionalized polydiarylfluorenes with the proportion from 10% to 40% and systematically investigated the impact of interchain hydrogen bonds on the polymer chain behavior either in solution or condensed state. Combining the results of DLS and rheological measurement, we concluded that the cross-linked interchain hydrogen bonds was facilitated to the chain entanglement and aggregation in solution, which resulted in solution gelation at the amide ratio of 30% and 40%. In addition, the hydrogen bonds are favourable for realizing the long-range-order, well-organized stacking structure of polymer chains, demonstrated by the pronounced diffractions peaks in the WAXS test. More importantly, we exploited a practical method for measuring the mechanical properties of CPs taking full advantage of the conventional tensile instrument, through preparing a transferable thin film assisted by a specially designed hollow holder. After introducing amide-containing side chains, the CPs films become more tough and stretchable enabled by the interchain hydrogen bonds, and the maximum tensile strain reached to 25% for P4. Finally, PLED devices based on these CPs films that showed comparable performance. We also achieved random laser emission from these thin films in slab waveguides structures with the threshold ranging from 0.52 *μ*J/cm^2^ to 1.38 *μ*J/cm^2^, and the random cavity obtained from the Fourier transform spectroscopy was about 30~60 *μ*m. These observations suggested that interchain hydrogen bonds would significantly promote the potential application of CPs in the field of flexible optoelectronic devices.

## 4. Materials and Methods

### 4.1. Materials and General Measurements

The detailed procedures for preparing the novel amide-containing monomer and polymerization of these novel copolymers were attached in supporting information. Variable temperature FT-IR experiments were carried out on a Thermo Fisher IS50 spectrophotometer equipment with a temperature controller. The samples were heated from 30°C to 200°C and collected every 10°C. The wide-angle X-ray scattering measurements were carried out using Anton Paar SAXSpoint 2.0, and the thin films were drop coated from 10 mg/mL toluene solution. The GIXRD data were obtained at beamline BL14B1 of the Shanghai Synchrotron Radiation Facility (SSRF). More details of general characteristic measurements also could be found in the supporting information.

### 4.2. Rheological Measurements

Their respective shear sweep viscosity measurements were performed with a DHR-2 rheometer (TA Instrument) with a cone plate attachment (diameter 40 mm, cone angle 0.1 rad, gap size 200 *μ*m). These copolymers were dissolved in chlorobenzene with a concentration of 10 mg mL^−1^.

### 4.3. Sample Fabrication for Tensile Test

A ~40 nm thick water soluble sacrificial layer of PEDOT: PSS (Clevios PVP AI 4083, Heraeus) was spin-coated on a precleaned glass substrate (5 mm × 10 mm) at 2500 rpm for 60 s and annealed at 120°C for 20 min. Subsequently, these copolymers tested in this work were dissolved in chlorobenzene at 50 mg mL^−1^ and spin-coated at 1500 rpm for 40 s, yielding a thick film about 800 nm. Then, the sample was immersed in deionized water and heated to 60°C in order to release the membrane from the rigid glass. After the membrane floated onto the water, we transfer the membrane to the PMMA hollow substrate and take it out from water carefully in order to prohibit fracture occurred. The detailed size information of the hollow substrate is shown in Figure S14. The thickness of these copolymer films were measured using a profilometer (KLA Tencor P-7).

### 4.4. Stress-Strain Tests

The sample was clamped to the tensile test configuration on a dynamic mechanical analyzer (DMA Q800, TA Instruments) for tensile tests. Before testing, the two edge connections of the hollow PMMA holder was melted using a soldering iron. The active area of the specimen was 5 mm × 2 mm (length × width). The stress-strain plots were obtained with a constant ramp applied force (0.005 N/min), and the displacement and applied force were measured independently.

### 4.5. Nanoindentation Tests

Nanoindentation measurements were recorded on Bruker's Hysitron TI Premier Nanomechanical Test Instrument with a Berkovitch diamond indenter. The radius of the Berkovitch-type diamond pyramid tip was <20 nm. Thin films for this test were spin-coated at quartz substrate from the solution of chlorobenzene (50 mg mL^−1^). Every sample was performed with three loading/unloading cycles, and the depth for each cycle was 60 nm, 120 nm, and 180 nm, respectively, in order to minimize the effect substrate. The values of the elastic modulus (*E*_r_) and hardness (*H*) were extracted from the force-depth (load-displacement) curves according to the Oliver-Pharr method, where a fit of the unloading curve is used to determine the stiffness, the contact depth, the reduced modulus of the system, and finally the modulus of the sample. The reduced modulus is defined as:

(1) $$\begin{array}{c} E_{\text{r}}=\frac{S\sqrt{\pi }}{2\sqrt{A}}, \end{array}$$

where *S* is the stiffness of the unloading curve and *A* is the projected contact area. The hardness is defined as the maximum indentation force divided by the resultant projected contact area at that load:

(2) $$\begin{array}{c} H=\frac{P_{\max}}{A}. \end{array}$$

Poisson's ratio varies between 0 and 0.5 for most materials, and it was assumed to be 0.20 for these conjugated materials in this work.

### 4.6. Fabrication and Characterization of PLEDs

All PLED devices were prepared and characterized following the process as follows. The ITO substrates were cleaned in an ultrasonic bath with detergent, acetone, isopropanol, and deionized water, dried in an oven at 120°C for 2 hours, and treated with ultraviolet ozone for 10 min before spin-coating. Firstly, a 40 nm thick PEDOT: PSS was spin-coated and then annealed at 120°C for 20 minutes. Then, the emitting layer was spin-coated from CB solution (10 mg/mL) and annealed at 120°C for 15 minutes in nitrogen-filled glovebox. Finally, the residue layers, such as 20 nm TPBi, 0.8 nm LiF, and 100 nm Al, were deposited by thermal evaporating at a pressure below 1 × 10^−5^ mbar. The *J* − *L* − *V* curves were recorded using a combination of a Keithley source meter (model 2602) and a luminance meter. The EL spectra of the devices were measured using a PR-655 spectrophotometer. All the measurements were taken in the ambient condition at room temperature.

### 4.7. Characterization of Optical Gain Properties

The configuration of planar waveguides was fabricated from CB solution onto quartz substrates by spin-coating, while the concentration was 20 mg/mL. The thickness of thin films were about 80 nm. The samples were optically pumped at 355 nm with the second harmonic of a femtosecond regenerative amplifier (Clark-MXR model CPA-1) delivering pulses of 150 fs duration at 1 kHz repetition rate. The pump beam was focused with a cylindrical lens and spatially filtered through an adjustable slit to create a 110 *μ*m × 3 mm excitation stripe on the sample. The photoluminescence arising from the edge of the waveguide was spectrally dispersed with a spectrometer (SP2500, Acton Research) equipped with a liquid nitrogen-cooled back-illuminated deep depletion CCD (Spec-10: 400BR, Princeton Instruments).

## Data Availability

All of the relevant data that support the findings in this work are available upon request from the corresponding author under reasonable request.

## Supplementary Materials

Supplementary Materials Scheme S1: synthesis procedure for P (ODPF-*co*-ADPF). Table S1: summary of the feed ratios and the calculated amide proportion of copolymers. Table S2: summary of the thermal, photophysical, and electrochemical properties of P1-P4. Table S3: summary of the tensile modulus and hardness measured by other methods. Figure S1: ^1^H-NMR and ^13^C-NMR spectra of monomer B. Figure S2: ^1^H-NMR of the copolymers in CDCl_3_. Figure S3: ^1^H-NMR of the copolymers in CDCl3 after added a few drops of MeOH-d_4_. Figure S4: GPC curves of P1-P4. Figure S5: TG and DSC curves. Figure S6: the varied temperature FT-IR spectra of P4. Figure S7: FT-IR spectra of P1-P4 compared with PODPF. Figure S8: The varied temperature FT-IR spectra in several heating and cooling cycles. Table S4: the mixed ratio of P2 and P3 solutions (10 mg/mL). Figure S9: the mixed solutions state after aging for several weeks. Figure S10: photographs of P3 and P4 aging in chlorobenzene. Figure S11: Comparison of the AFM height images of P1-P4. Figure S12: GIXRD of the drop-coated films. Figure S13: XRD curves of P3 and P4. Figure S14: The size information of holder and images during the tensile process. Figure S15: photos of the tensile film under optical microscope with the FOE method. Figure S16: optical microscope photos of the conjugated polymer films under strain. Figure S17: TCSPC measurement. Figure S18: CV curves of these copolymers. Figure S19: the Fourier transform spectra of random laser emission spectra.
