# Supplementary material for: Unveiling the Effects of Interchain Hydrogen Bonds on Solution Gelation and Mechanical Properties of Diarylfluorene-Based Semiconductor Polymers
Source: Research (Wash D C). 2020 Sep 30;2020:3405826. doi: 10.34133/2020/3405826 (PMC7545494; doi:10.34133/2020/3405826)
Supplement: Supplementary Materials — Scheme S1: synthesis procedure for P (ODPF-co-ADPF). Table S1: summary of the feed ratios and the calculated amide proportion of copolymers. Table S2: summary of the thermal, photophysical, and electrochemical properties of P1-P4. Table S3: summary of the tensile modulus and hardness measured by other methods. Figure S1: 1H-NMR and 13C-NMR spectra of monomer B. Figure S2: 1H-NMR of the copolymers in CDCl3. Figure S3: 1H-NMR of the copolymers in CDCl3 after added a few drops of MeOH-d4. Figure S4: GPC curves of P1-P4. Figure S5: TG and DSC curves. Figure S6: the varied temperature FT-IR spectra of P4. Figure S7: FT-IR spectra of P1-P4 compared with PODPF. Figure S8: The varied temperature FT-IR spectra in several heating and cooling cycles. Table S4: the mixed ratio of P2 and P3 solutions (10 mg/mL). Figure S9: the mixed solutions state after aging for several weeks. Figure S10: photographs of P3 and P4 aging in chlorobenzene. Figure S11: Comparison of the AFM height images of P1-P4. Figure S12: GIXRD of the drop-coated films. Figure S13: XRD curves of P3 and P4. Figure S14: The size information of holder and images during the tensile process. Figure S15: photos of the tensile film under optical microscope with the FOE method. Figure S16: optical microscope photos of the conjugated polymer films under strain. Figure S17: TCSPC measurement. Figure S18: CV curves of these copolymers. Figure S19: the Fourier transform spectra of random laser emission spectra. [file 3405826.f1.docx]

**Supporting Information**

Unveiling the Effects of Interchain Hydrogen Bonds on Solution Gelation and Mechanical Properties of Diarylfluorene-based Semiconductor Polymers

Lubing Bai^1^, Yamin Han^1^, Chen Sun^1^, Xiang An^1^, Chuanxin Wei^1^, Wei Liu^4^, Man Xu^2^, Lili Sun^1^, Ning Sun^1^, Mengna Yu^2^, He Zhang^2^, Qi Wei^4^, Chunxiang Xu^4^, Yingguo Yang,^5^ Tianshi Qin,^1^ Linghai Xie^2^, Jinyi Lin^1,4^* and Wei Huang^1,2,4^*

^1^Center for Supramolecular Optoelectronics (CSO), Key Laboratory of Flexible Electronics (KLOFE) and Institute of Advanced Materials (IAM), Nanjing Tech University (NanjingTech), 30 South Puzhu Road, Nanjing 211816, China.

^2^Center for Molecular Systems and Organic Devices (CMSOD), Key Laboratory for Organic Electronics and Information Displays & Institute of Advanced Materials (IAM), Nanjing University of Posts & Telecommunications, 9 Wenyuan Road, Nanjing 210023, China

^3^State Key Laboratory of Bioelectronics, School of Electronic Science and Medical Engineering, Southeast University, Nanjing 210096, China.

^4^Shaanxi Institute of Flexible Electronics (SIFE), Northwestern Polytechnical University (NPU), 127 West Youyi Road, Xi'an710072, Shaanxi, China.

^5^Shanghai Synchrotron Radiation Facility (SSRF), Zhangjiang Lab, Shanghai Advanced Research Institute, Chinese Academy of Sciences, 239 Zhangheng Road, Shanghai 201204, China

Lubing Bai and Yamin Han contributed equally to this work.

Correspondence and requests for materials should be addressed to J. L. (email: iamjylin@njtech.edu.cn) or W. H. (email: iamwhuang@nwpu.edu.cn)

**Contents**

- **Scheme S1**. Synthesis procedure for P(ODPF-co-ADPF)
- **Table S1.** Summary of the feed ratios and the calculated amide proportion of copolymers.
- **Table S2.** Summary of the thermal, photophysical, electrochemical properties of P1-P4.
- **Table S3.** Summarize of the tensile modulus and hardness measured by other methods.
- **Figure S1.** ^1^H-NMR and ^13^C-NMR spectra of monomer B.
- **Figure S2.** ^1^H-NMR of the copolymers in CDCl_3_.
- **Figure S3.** ^1^H-NMR of the copolymers in CDCl_3_ after added a few drops of MeOH-*d*^4^.
- **Figure S4.** GPC curves of P1-P4.
- **Figure S5.** Tg and DSC curves.
- **Figure S6.** The varied temperature FT-IR spectra of P4.
- **Figure S7.** FT-IR spectra of P1-P4 compared with PODPF.
- **Figure S8.** The varied temperature FT-IR spectra in several heating and cooling cycles.
- **Table S4.** The mixed ratio of P2 and P3 solutions (10 mg/mL).
- **Figure S9.** The mixed solutions state after aging for several weeks.
- **Figure S10.** Photographs of P3 and P4 aging in chlorobenzene.
- **Figure S11.** Comparation of the AFM height images of P1-P4.
- **Figure S12.** GIXRD of the drop-coated films.
- **Figure S13.** XRD curves of P3 and P4.
- **Figure S14.** The size information of holder and images during the tensile process.
- **Figure S15.** Photos of tensile the film under optical microscope with the FOE method.
- **Figure S16.** Optical microscope photos of the conjugated polymer films under strain.
- **Figure S17.** TCSPC measurment.
- **Figure S18.** CV curves of these copolymers.
- **Figure S19.** The Fourier transform spectra of random laser emission spectra.

**Reagents and materials:**

Reagent and catalysts used in the process were commercially available. 2,2'-bipyridine, 1,5-cyclooctadiene (COD) and bis(1,5-cyclooctadiene)nickel(0) (Ni(COD)2) were purchased from Sigma-Aldrich. 6-bromohexanoyl chloride, butan-1-amine, boron trifluoride diethyl etherate complex (BF_3_•Et_2_O) and triethylamine (Et_3_N) were purchased from Alfa Aesar. Dimethylformamide (DMF) was dried over calcium hydride (CaH) and distilled under a dry nitrogen atmosphere immediately prior to use. Toluene was dried over Na and distilled under a dry nitrogen atmosphere immediately prior to use.

**General Characterization**: The number-average molecular weights were estimated by gel permeation chromatography (GPC) analysis using DMF as the eluent and linear polystyrene as the standard. UV-visible absorption spectra were taken with a Shimadzu UV-1750 spectrometer at room temperature, and photoluminescence spectra were measured using Hitachi F-4600. Thermogravimetric analysis (TGA) was acquired by TGA2 (Mettler Instruments). Differential scanning calorimetry (DSC) data was measured by DSC214 Polyma (NETZSCH Instruments) with the measured temperature from 25 to 300 ^o^C at a rate of 10 ^o^C/min. Cyclic voltammetric (CV) studies were taken using an CHI660C Electrochemical Work station in a three-electrode cell, including a platinum sheet working electrode, a platinum wire counter electrode and a silver/silver nitrate (Ag/Ag^+^) reference electrode. The film morphologies of polymer films were measured with AFM in tapping mode (Bruker’s Dimension Icon). The dynamic light scattering (DLS) measurements were carried out using an ALV/CGS-3. The hydrodynamic radius distribution was obtained using the Stokes-Einstein equation, *R*_h_ = *k*_B_*T*/(6*πηD*), where *k*_B_ is the Boltzmann constant, *T* is absolute temperature, *D* is the diffusion coefficient, and *η* is solvent viscosity. We take the X-ray diffraction (XRD) diffraction measurements by Smartlab (3KW). FT-IR spectra were recorded on a Bruker Tensor 27 FT-IR spectrophotometer.

**Synthesis procedure for the novel monomer B and copolymers:**


**Scheme S1**. Synthesis procedure for P(ODPF-co-ADPF).

**Synthesis procedures for monomer B:**

4,4'-dibromo-2'-(hydroxy diphenylmethyl)biphenyl-2-ol (2OH) and the monomer A was synthesized according to our previous report.^6^ First, we prepared the reactant of Br-NH in a round-bottom flask (50 mL) equipped with a magnetic stir bar. The reactants of butan-1-amine (0.34 g, 4.68 mmol) and Et_3_N (0.8 g, 8 mmol) was dissolved in anhydrous DCM (15 mL) and stirred under the ice bath. Then, 6-bromohexanoyl chloride (1 g, 4.68 mmol) was dropped in the flask and kept the reaction stirred for 5 hours. After the reaction completed, the solvent of DCM and excess Et_3_N was removed under reduced pressure and the residue was redissolved in acetone (20 mL). Then, 2OH (1.5 g, 3 mmol) was added in the flask and stirred at room temperature for 24 hours, then the solvent of acetone was removed under reduced pressure and the residue was redissolved in DCM (15 mL), followed by adding BF_3_•Et_2_O (0.5 mL) to the flask. At last, the reaction was poured into water and extracted with dichloromethane three times. The organic phase was dried with Na_2_SO_4_ and the solvent was removed in reduced pressure. The crude product was purified by flash column chromatography (silica gel, petroleum ether/ethyl acetate 4:1) to give the target compound as a white solid (1.2 g, 60%). ^1^H-NMR (CDCl_3_): δ 7.92 (d, 1H), 7.48 (d, 1H), 7.45 (d, 1H), 7.26-7.22 (m, 6H), 7.13 (dd, 4H), 7.09 (d, 1H), 6.97 (d, 1H), 5.40 (s, 1H), 4.13 (t, 2H), 3.25 (dd, 2H), 2.22 (t, 2H), 2.05-1.92 (m, 2H), 1.84-1.72 (m, 2H), 1.52-1.41 (m, 2H), 1.39-1.27 (m, 4H), 0.91 (t, 3H). ^13^C-NMR (CDCl_3_): δ 172.59, 155.46 , 154.09, 152.47, 144.48, 137.64, 130.88, 128.78, 128.51, 128.08, 127.13, 126.25, 125.22, 122.14, 121.45, 120.90, 113.89, 68.30, 65.91, 39.29, 36.72, 31.76, 29.02, 25.97, 25.47, 20.10, 13.78.

**General procedures for Yamamoto copolymerization:**

The feed ratios of monomer A and B for polymerization were summarized in Table S1 and the detailed procedure of Yamamoto copolymerization was conducted as follows^7^: Monomer A and B, Ni(COD)_2_ (1 g), 2,2'-bipyridine (0.5 g) were added in a 25 mL Schlenk flask and was degassed with N_2_ for three times. Then, COD (0.5 mL) were added to the flask followed by the injection of degassed DMF (15 mL) and stirred at 75 ^o^C for 30 min. Then the degassed toluene (10 mL) was added and adjusted the temperature to 85 ^o^C. The reaction mixture was stirred at dark for 72 h. At the end of the polymerization, bromobenzene were added to the flask and refluxing for 6 h. After the reaction cooled down to room temperature, the complex was filtered and the organic phase was purified by flash column chromatography (Al_2_O_3_) with DCM as mobile phase. After the solvent was concentrated to viscous in reduced pressure, the copolymers were received by precipitation in methanol. At last, the resulted polymers were washed by Soxhlet extraction with acetone and hexane to remove oligomers and catalyst residues. The copolymer powder was dried under vacuum and used for this study (yields of 70~80%).

**Table S1.** Summary of the feed ratios and the calculated amide proportion of copolymers.

| polymers | A | B | Amide% (theoretical) | Amide% (calculated) |
| --- | --- | --- | --- | --- |
| P1 | 0.43 g (0.72 mmol) | 0.05 g (0.08 mmol) | 10% | 10% |
| P2 | 0.39 g (0.64 mmol) | 0.11 g (0.16 mmol) | 20% | 20% |
| P3 | 0.34 g (0.56 mmol) | 0.16 g (0.24 mmol) | 30% | 29% |
| P4 | 0.29 g (0.48 mmol) | 0.21 g 0.32 mmol | 40% | 40% |

**Table S2.** Summary of the thermal, photophysical, electrochemical properties of P1-P4.

|  | *M*_n_ (kDa) | PDI | *T*_g_ (^o^C) | *T*_d_  (^o^C) | Solution | | Film | | PLQY (%) | HOMO （eV） |
| --- | --- | --- | --- | --- | --- | --- | --- | --- | --- | --- |
|  |  |  |  |  | λ_abs_ | λ_em_ | λ_abs_ | λ_em_ |  |  |
| P1 | 14.3 | 2.0 | 172 | 410 | 396 | 431, 454 | 389 | 441, 468 | 34 ± 1 | -5.91 |
| P2 | 12.5 | 1.8 | 170 | 406 | 395 | 429, 453 | 390 | 439, 464 | 42 ± 1 | -5.84 |
| P3 | 12.0 | 1.9 | 166 | 403 | 393 | 429, 454 | 392 | 438, 460 | 37 ± 1 | -5.91 |
| P4 | 18.6 | 2.3 | 172 | 405 | 393 | 427, 454 | 396 | 439, 463 | 38 ± 1 | -5.92 |

**Table S3.** Summarize of the tensile modulus and hardness reported in other works.

| Polymer | | M_n_ (kDa) | Methods | Tensile (Young’s) modulus (GPa) | Hardness | Reference |
| --- | --- | --- | --- | --- | --- | --- |
| P3HT | | 15 | FOW*^a^* | 0.203 ± 0.014 | NA | 1 |
|  |  | 40 |  | 0.263 ± 0.015 | NA |  |
|  |  | 63 |  | 0.261 ± 0.020 | NA |  |
|  |  | 80 |  | 0.270 ± 0.012 | NA |  |
| P3HT | | 15 | FOE*^b^* | 0.71 ± 0.19 | NA |  |
|  |  | 40 |  | 1.79 ± 0.12 | NA |  |
|  |  | 63 |  | 1.46 ± 0.22 | NA |  |
|  |  | 80 |  | 0.92 ± 0.18 | NA |  |
| P3HT | | NA | FLOTE*^c^* | 0.18 | NA | 2 |
| RR-controlled P3HT | RR-98 | 20.3 | FOW | 0.287 ± 0.019 | NA | 3 |
|  | RR-86 | 20.8 |  | 0.11 ± 0.005 | NA |  |
|  | RR-80 | 13.6 |  | 0.068 ± 0.009 | NA |  |
|  | RR-75 | 12.3 |  | 0.013 ± 0.002 | NA |  |
| DPP-based polymers | P1-0% | 46.9 | FOW | 0.203 | NA | 4 |
|  | P2-5% | 36.5 |  | 0.17 | NA |  |
|  | P3-10% | 37.6 |  | 0.115 | NA |  |
|  | P4-20% | 10.1 |  | 0.106 | NA |  |
| F6 | | 7 | Nanoindentation | 19.9 ± 3.3 | 0.37 ± 0.07 | 5 |
| FC6 | | 10 |  | 12.3 ± 2.5 | 0.14 ± 0.04 |  |
| FOC6 | | 50 |  | 18.6 ± 1.6 | 0.57 ± 0.05 |  |
| FOC10 | | 14 |  | 7.3 ± 10.3 | 0.11 ± 0.01 |  |
| FNA | | 7.3 |  | 12.2 ± 1.7 | 0.53 ± 0.11 |  |
| FBZN | | 18 |  | 8.0 ± 0.4 | 0.40 ± 0.04 |  |
| FCN | | 48.1 |  | 10.5 ± 0.4 | 0.60 ± 0.03 |  |
| Fester | | 22.8 |  | 10.1 ± 0.3 | 0.66 ± 0.02 |  |
| FNEt | | 22 |  | 12.6 ± 0.6 | 0.74 ± 0.03 |  |
| FCBZ | | 9 |  | 8.2 ± 0.7 | 0.37 ± 0.03 |  |

*^a^*The conjugated polymer film is supported by water (Film-on-water). *^b^*The conjugated polymer film is supported by elastomer (Film-on-elastomer). *^c^*Film laminated on thin elastomer.


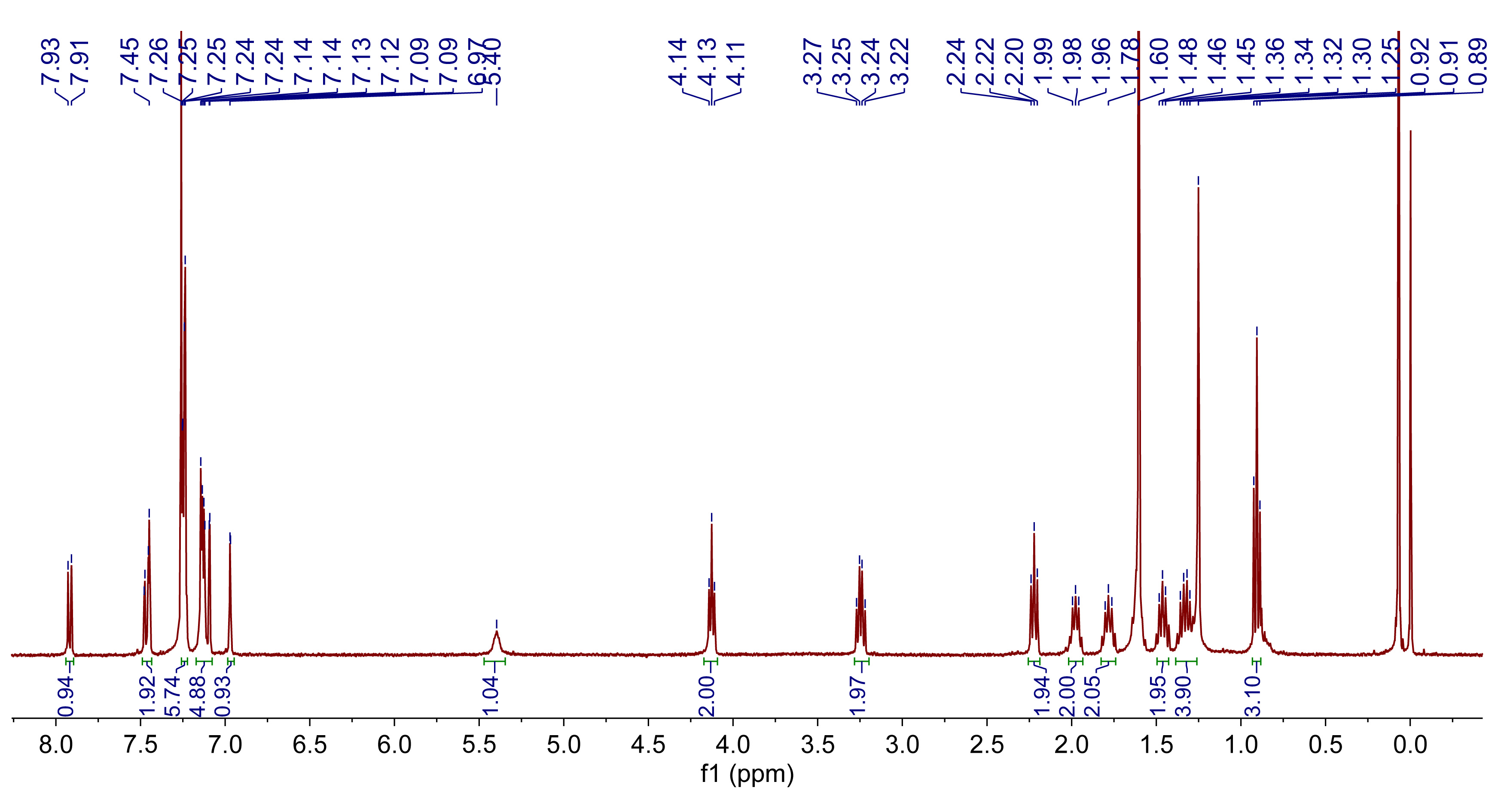


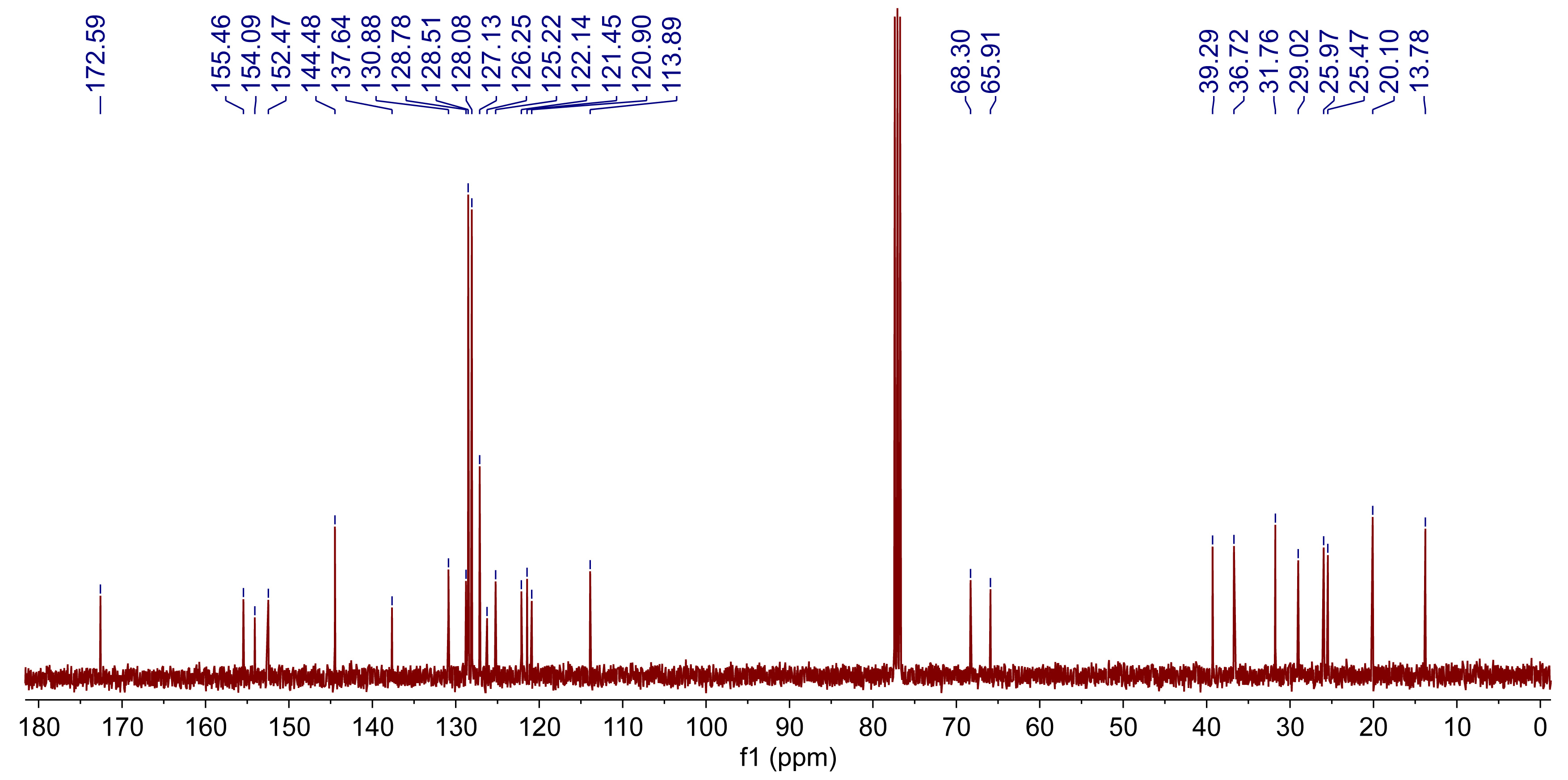


**Figure S1.** ^1^H-NMR and ^13^C-NMR spectra of monomer B.


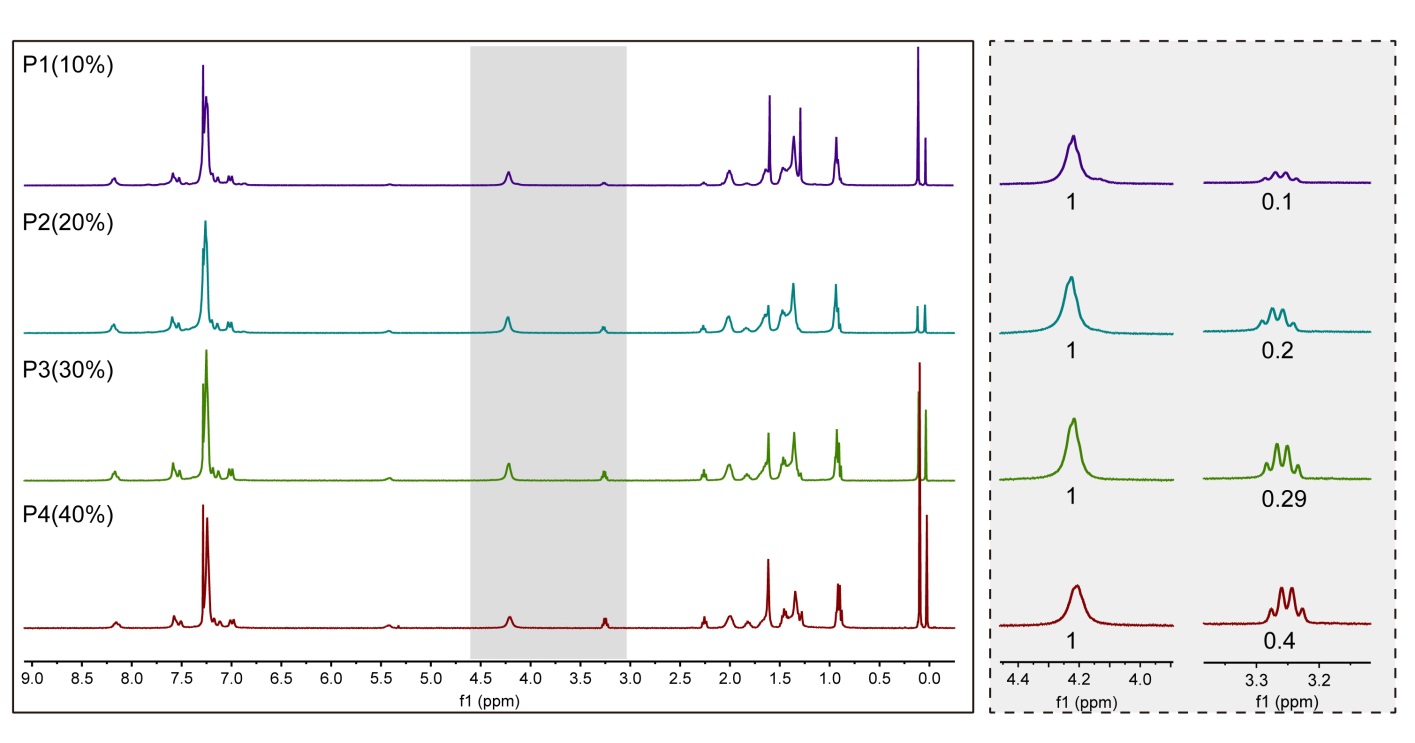


**Figure S2.** ^1^H-NMR of P1(10%), P2(20%), P3(30%) and P4(40%) in CDCl_3_. Right panel is the feature signals of protons near the ether linkage (4.22 ppm) and the carbonyl of amide group (3.2-3.3 ppm)


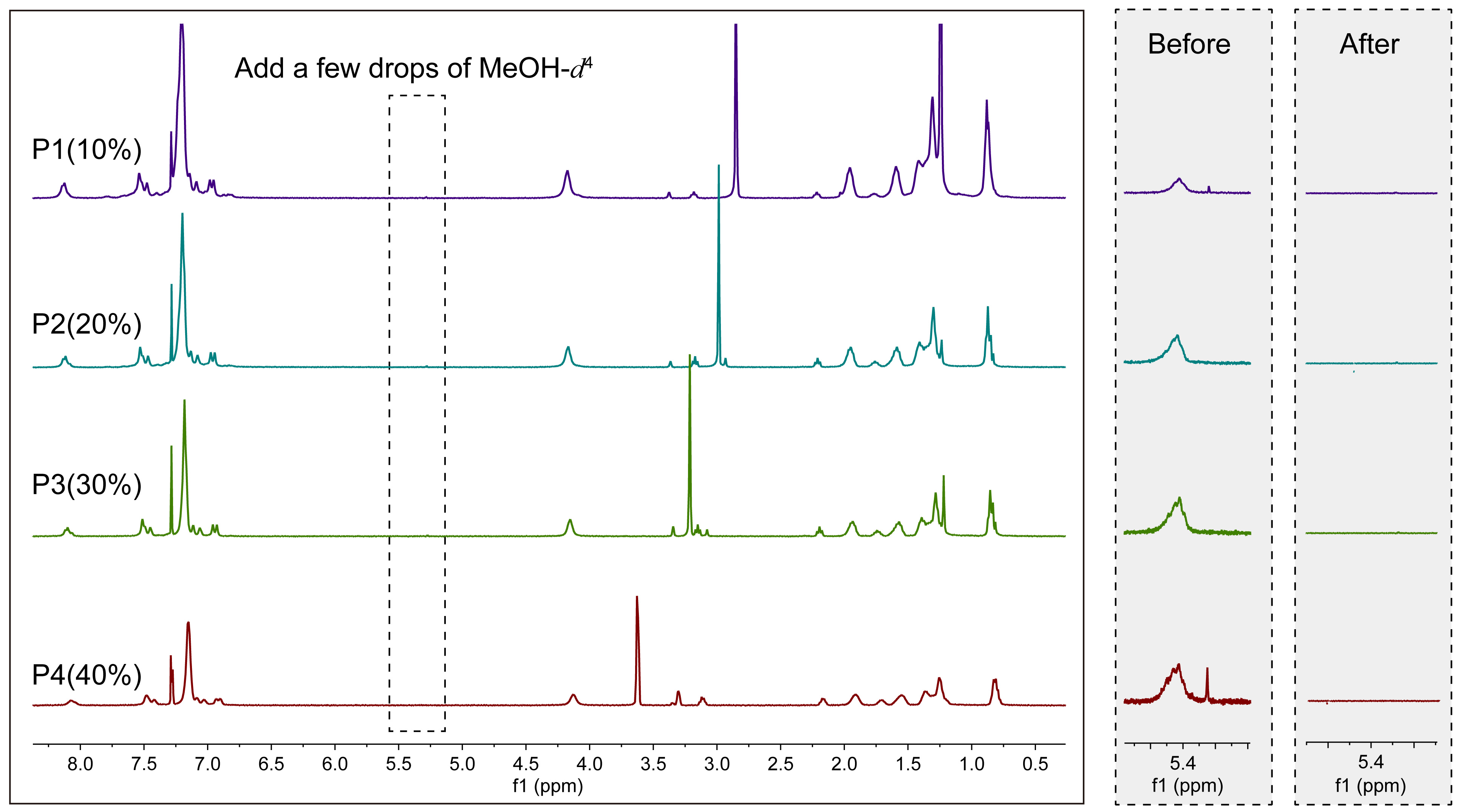


**Figure S3.** ^1^H-NMR of the P1-P4 after added a few drops of MeOH-*d*^4^. Right panels are the protons signals of amide groups in CDCl_3_ before and after adding MeOH-*d*^4^.

**Figure S4.** GPC curves of P1-P4 using DMF as the eluent.

**
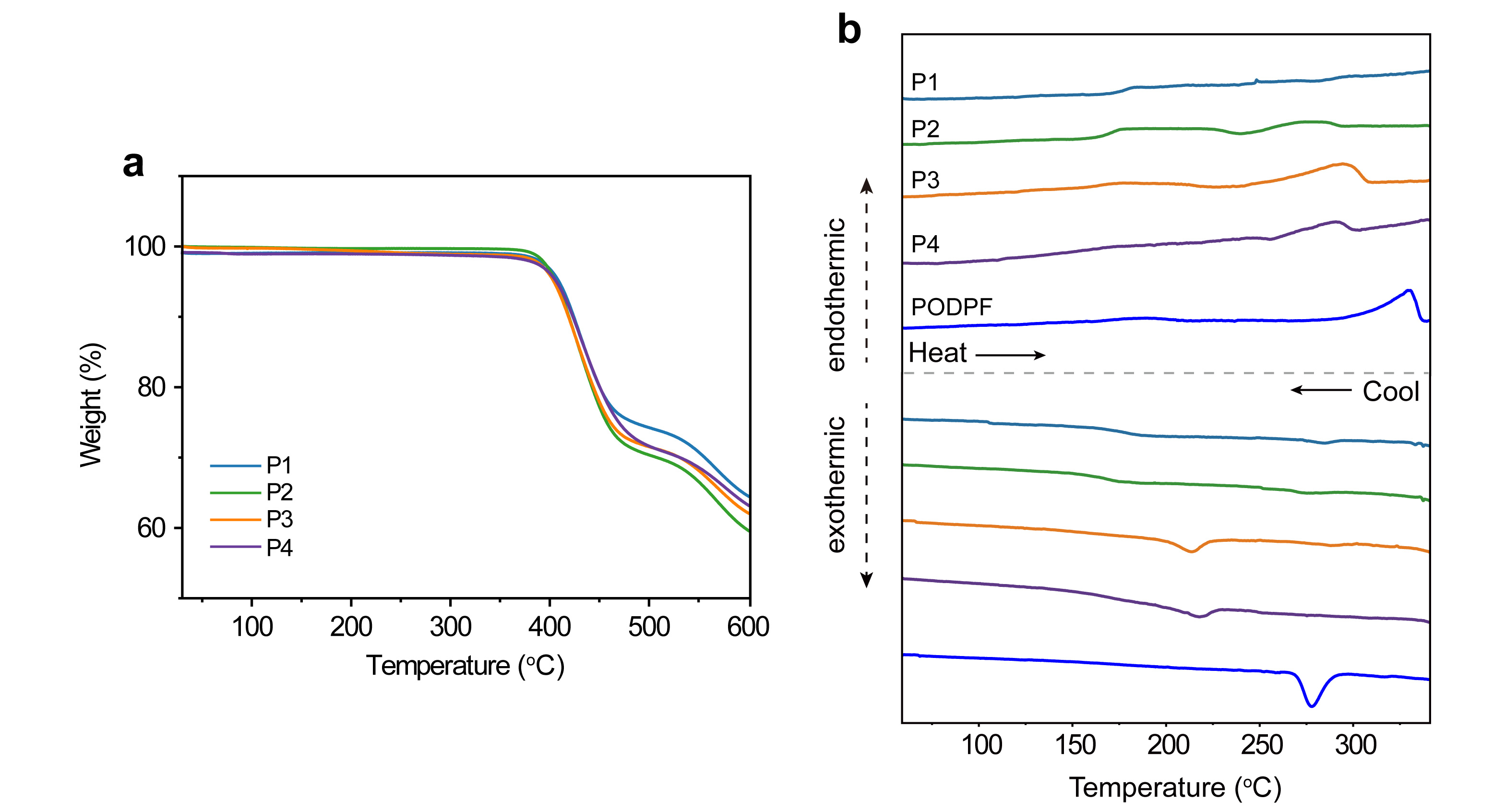
**

**Figure S5.** (a) Thermogravimetric (TG) and (b) DSC curves of P1, P2, P3 and P4. Heating rate was 10 K/min under nitrogen atmosphere.


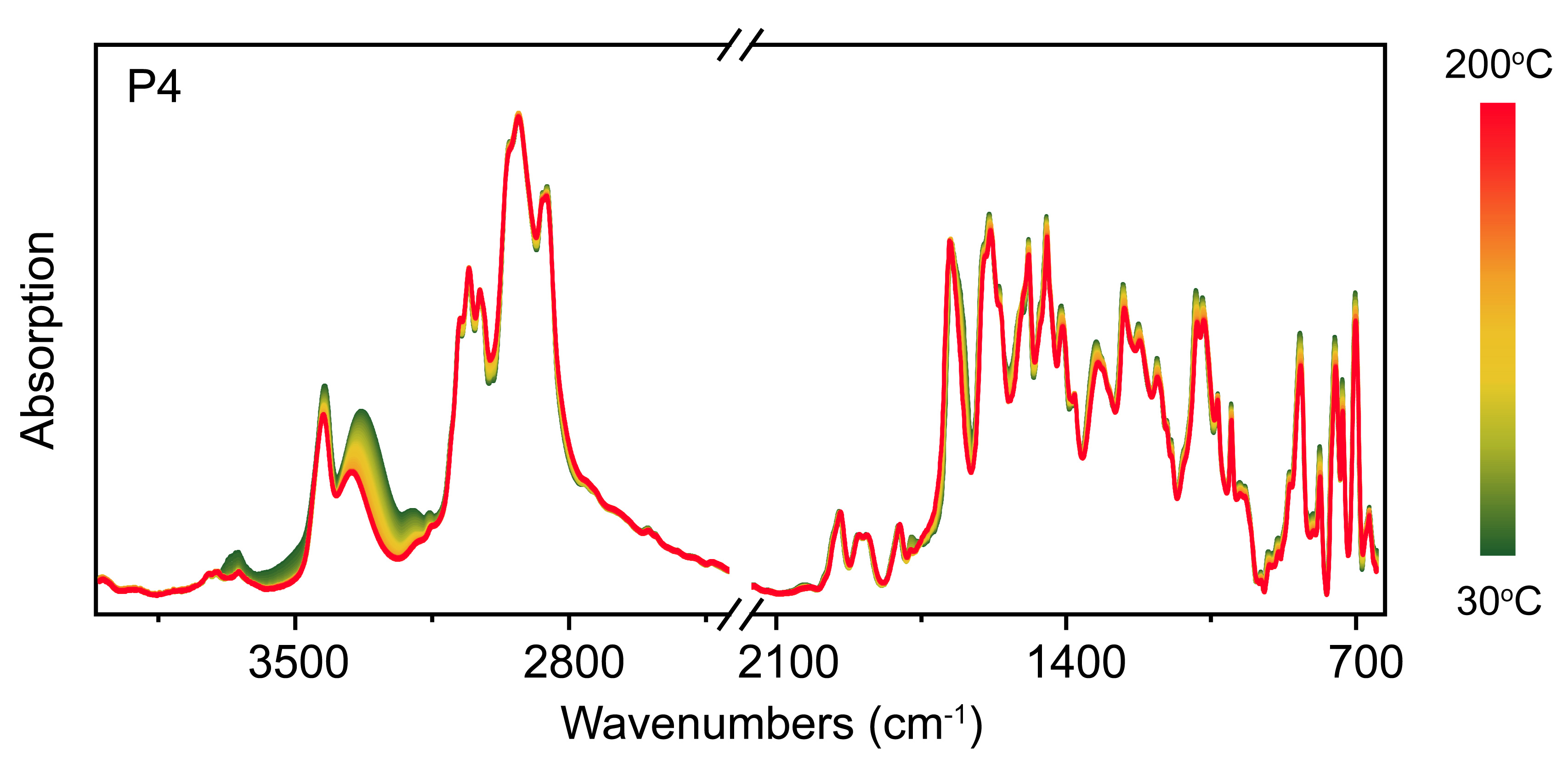


**Figure S6.** The whole spectra of varied temperature FT-IR regarded P4 as the representative sample.

**
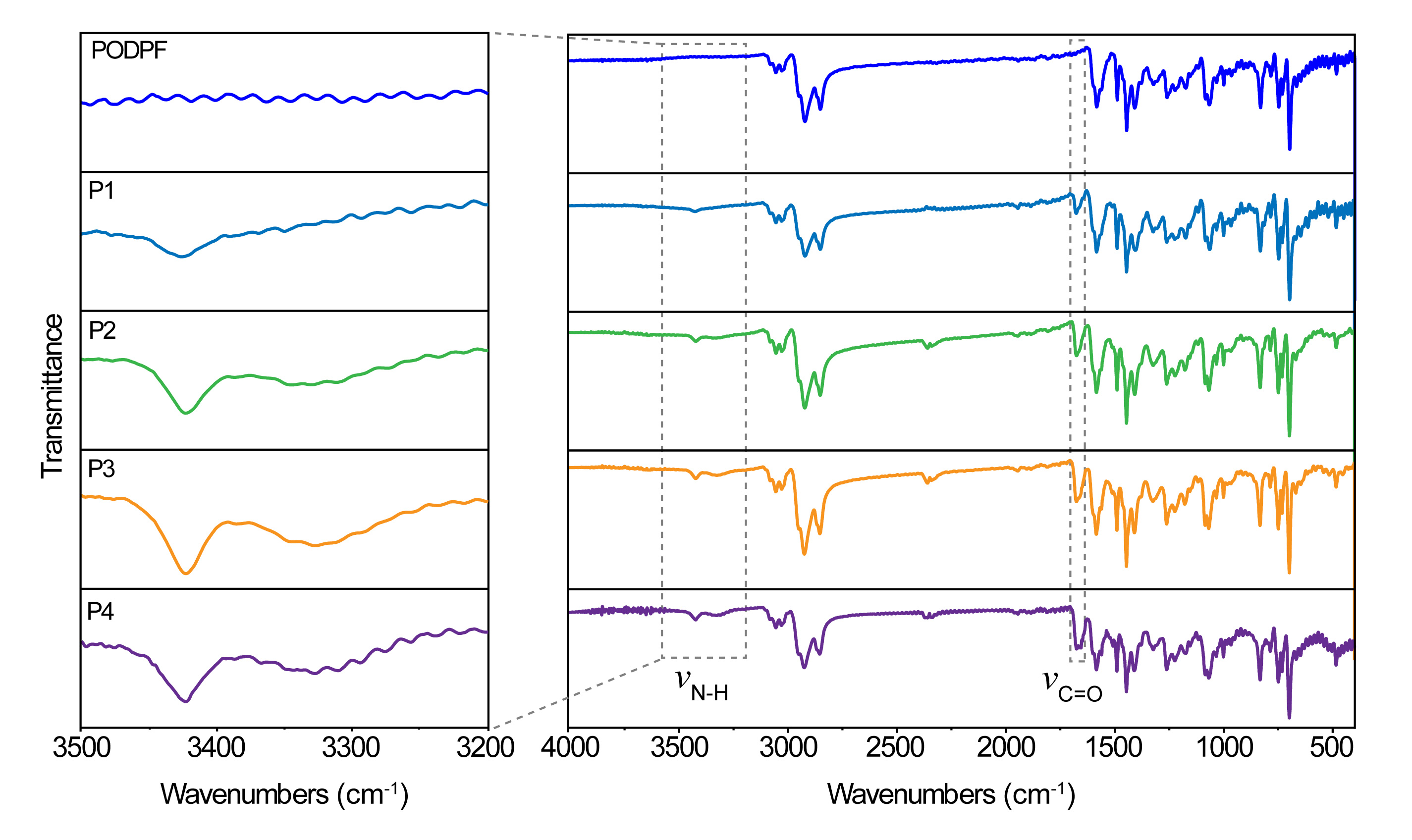
**

**Figure S7.** FT-IR spectra of P1-P4 compared with PODPF, of which the side chains are alkyl chains. Chemical structure of PODPF was shown in reference 1. It is obviously that there are two differences in the FT-IR spectra: one is at the range of 1640~1700 cm^-1^, and these peaks are assigned to the stretching mode of C=O; another is at the range of 3300~3500 cm^-1^, and these peaks are assigned to the stretching mode of N-H.


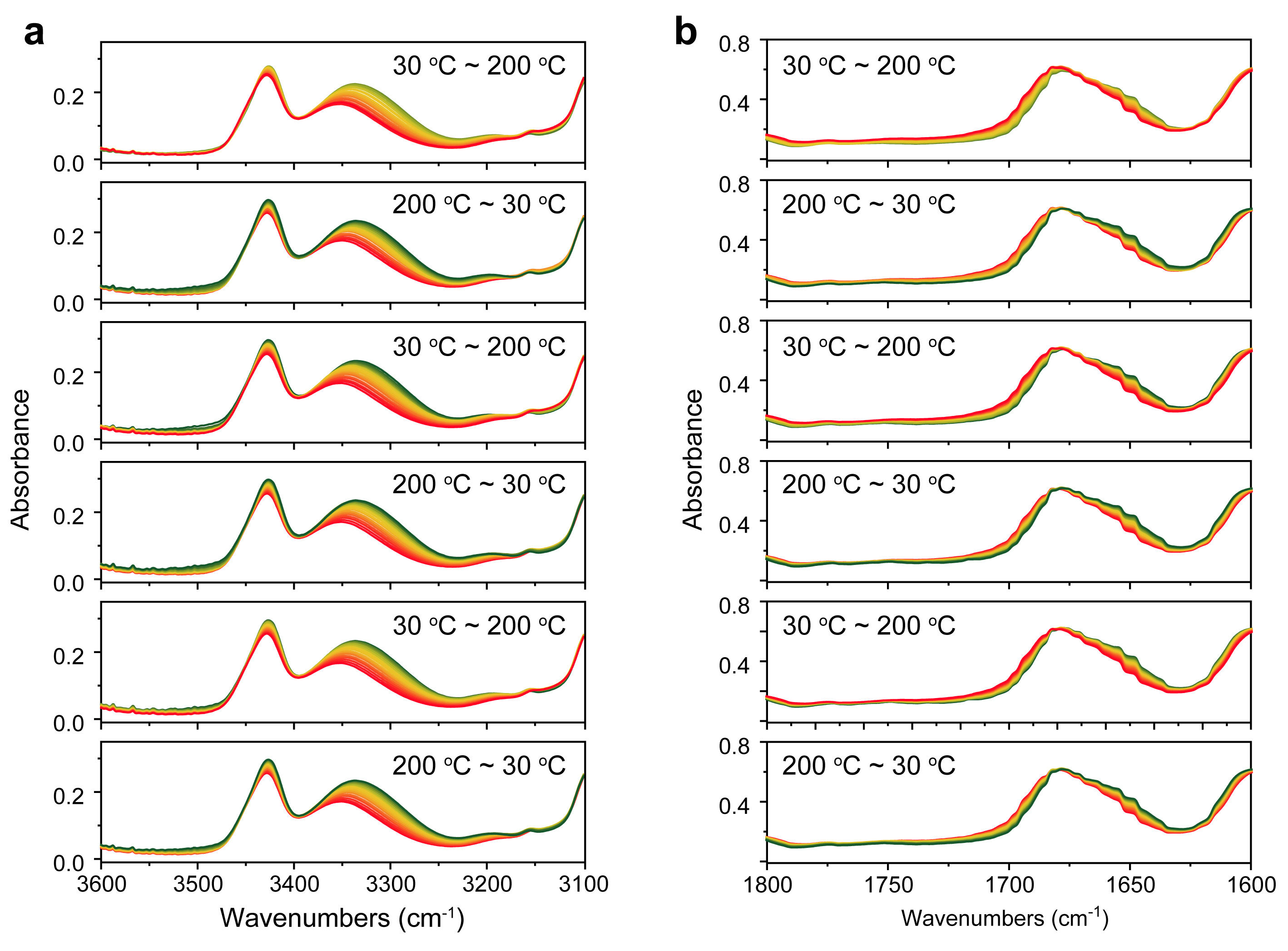


**Figure S8.** The varied temperature FT-IR spectra of P4 in several heating and cooling cycles. (a) The spectra region of 3100-3600 cm^-1^ corresponded to N-H vibrations. (b) The spectra region of 1600-1700 cm^-1^ corresponded to C=O vibrations.

**Table S4**. The mixed ratio of P2 and P3 solutions (10 mg/mL).

| Sample | P2(20%) | P3(30%) | Amide (%) |  |
| --- | --- | --- | --- | --- |
| 1 | 0.5 mL | 0 | 20% | Solution |
| 2 | 0.375 mL | 0.125 mL | 22.5% | Solution |
| 3 | 0.25 mL | 0.25 mL | 25% | Solution |
| 4 | 0.125 mL | 0.375 mL | 27.5% | Gelation |
| 5 | 0 | 0.5 mL | 30% | Gelation |


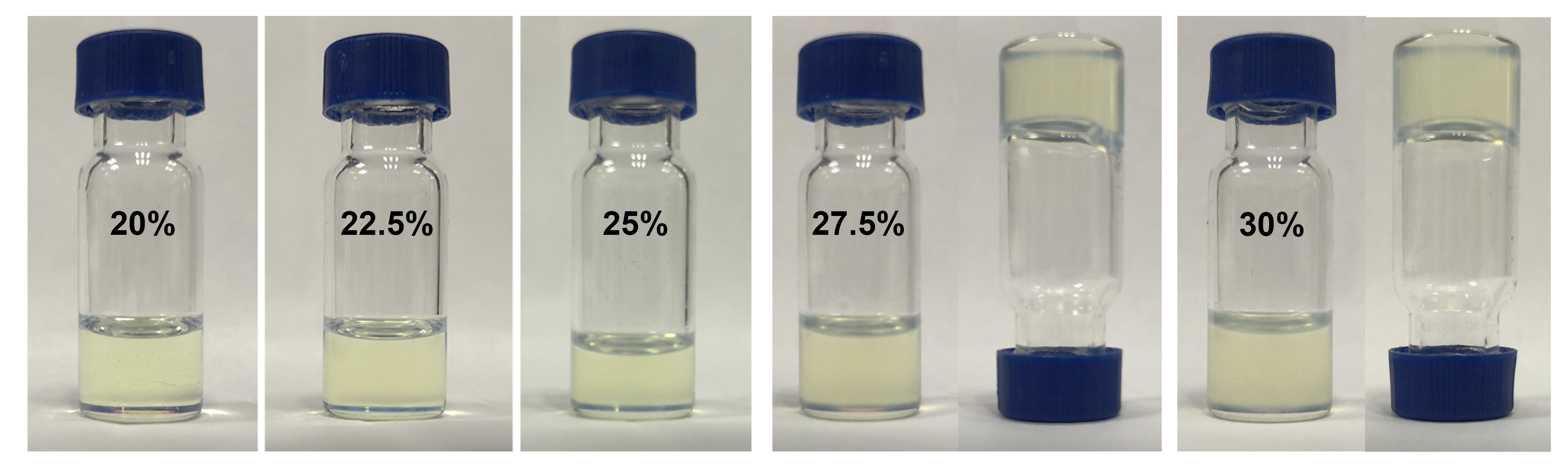


**Figure S9**. The mixed solutions state after aging for several weeks.

**
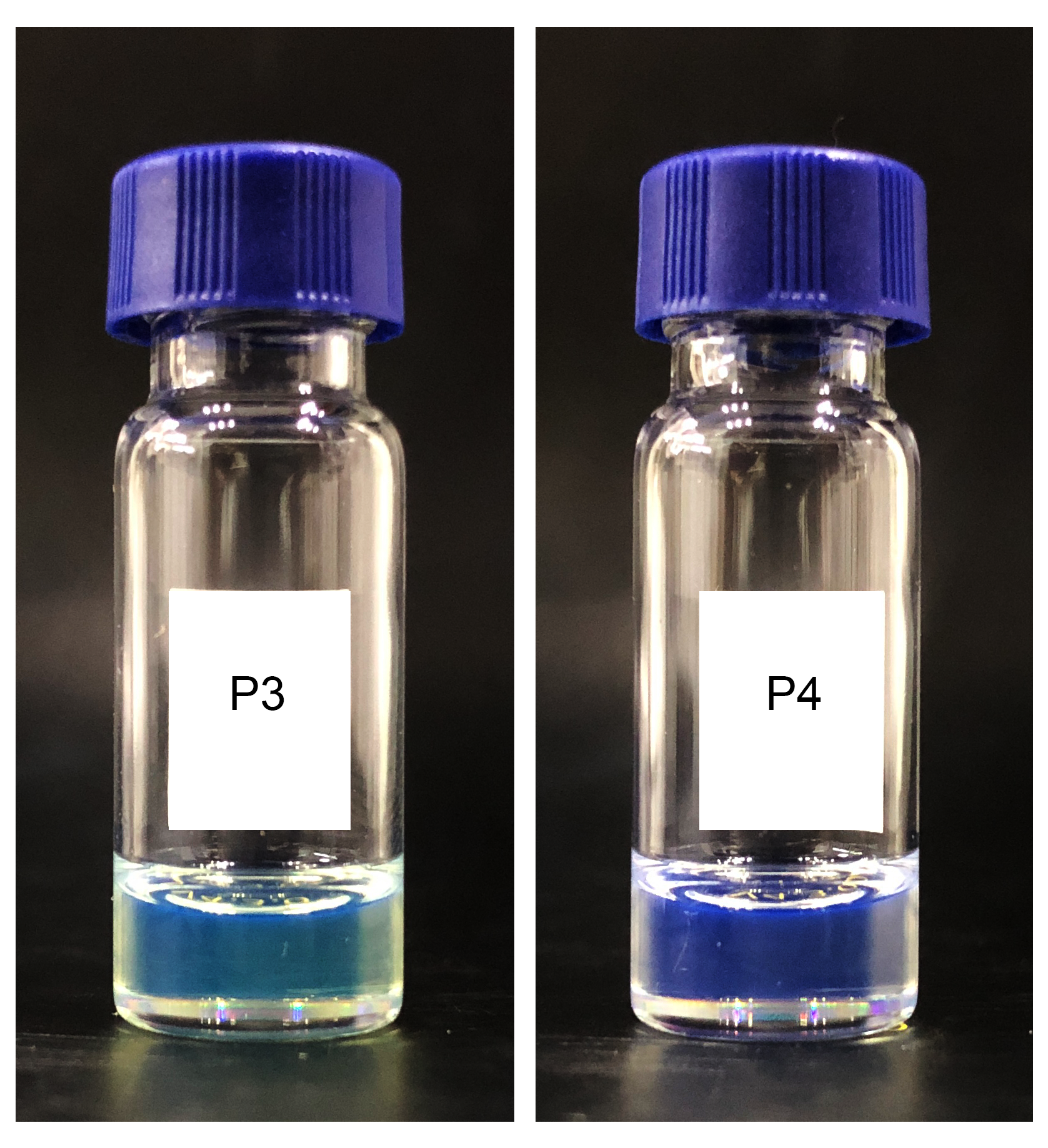
**

**Figure S10.** Photographs of P3 and P4 dissolved in chlorobenzene solutions with a concentration of 10 mg/mL after aging at room temperature for several weeks.

**
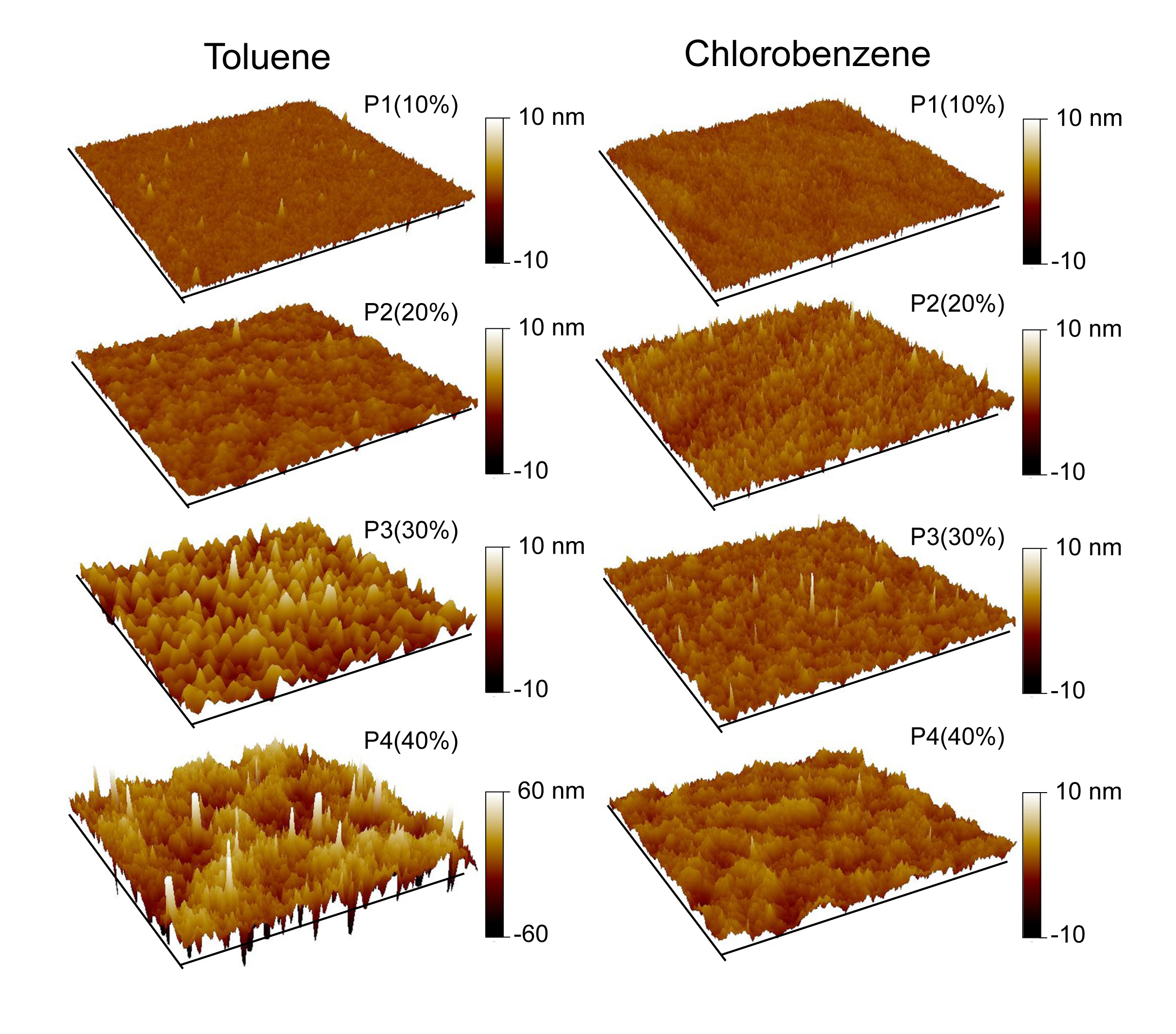
**

**Figure S11.** Comparation of the height images of P1-P4 films spin coated from toluene (left) and chlorobenzene (right) solutions (10 mg/mL), tested at the tapping mode of atomic force microscopy (AFM).

**
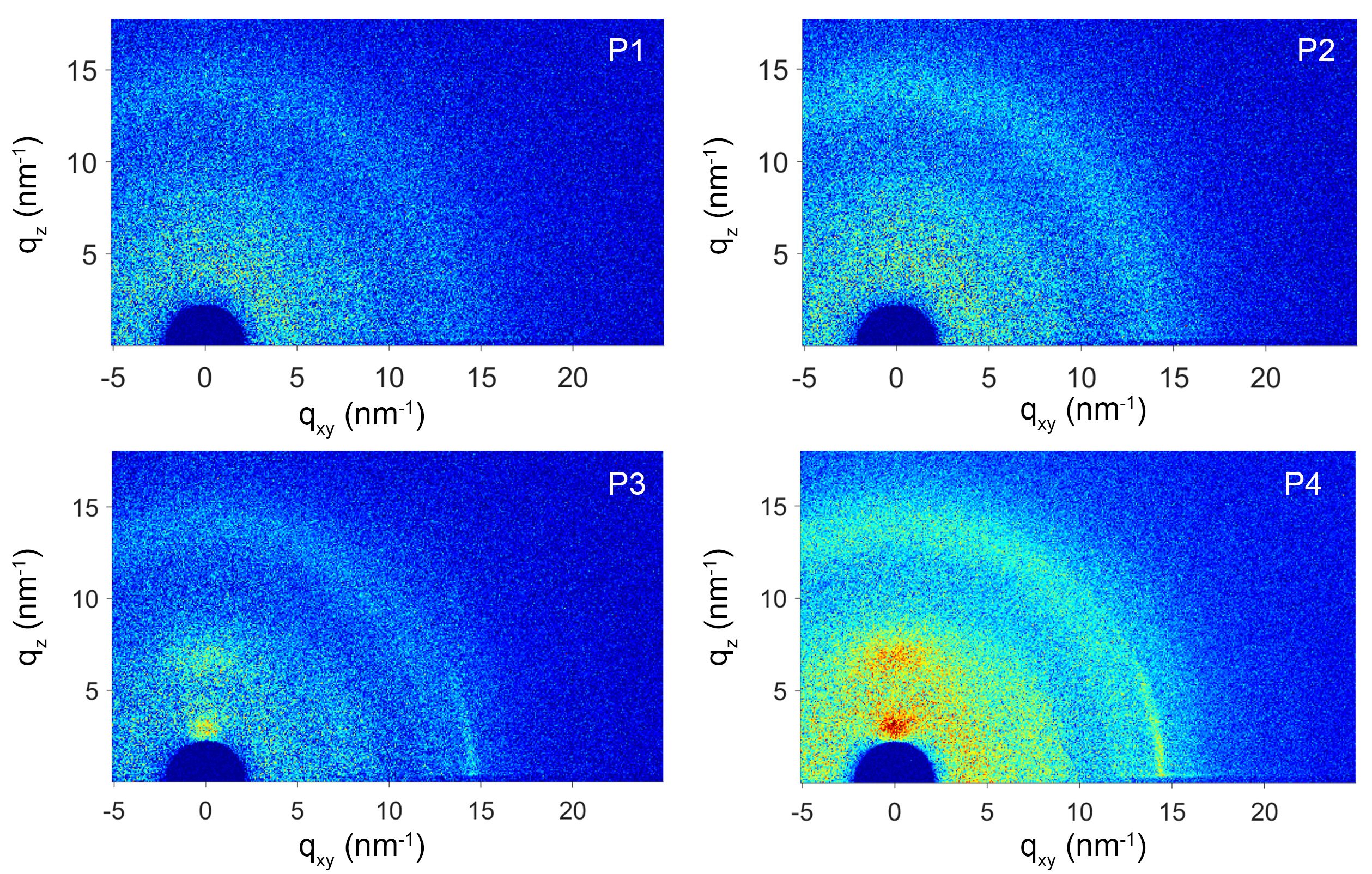
**

**Figure S12.** Grazing-incidence X-ray diffraction (GIXRD) of the drop-coated films, samples were made from toluene solution at a concentration of 1 mg/mL.

**
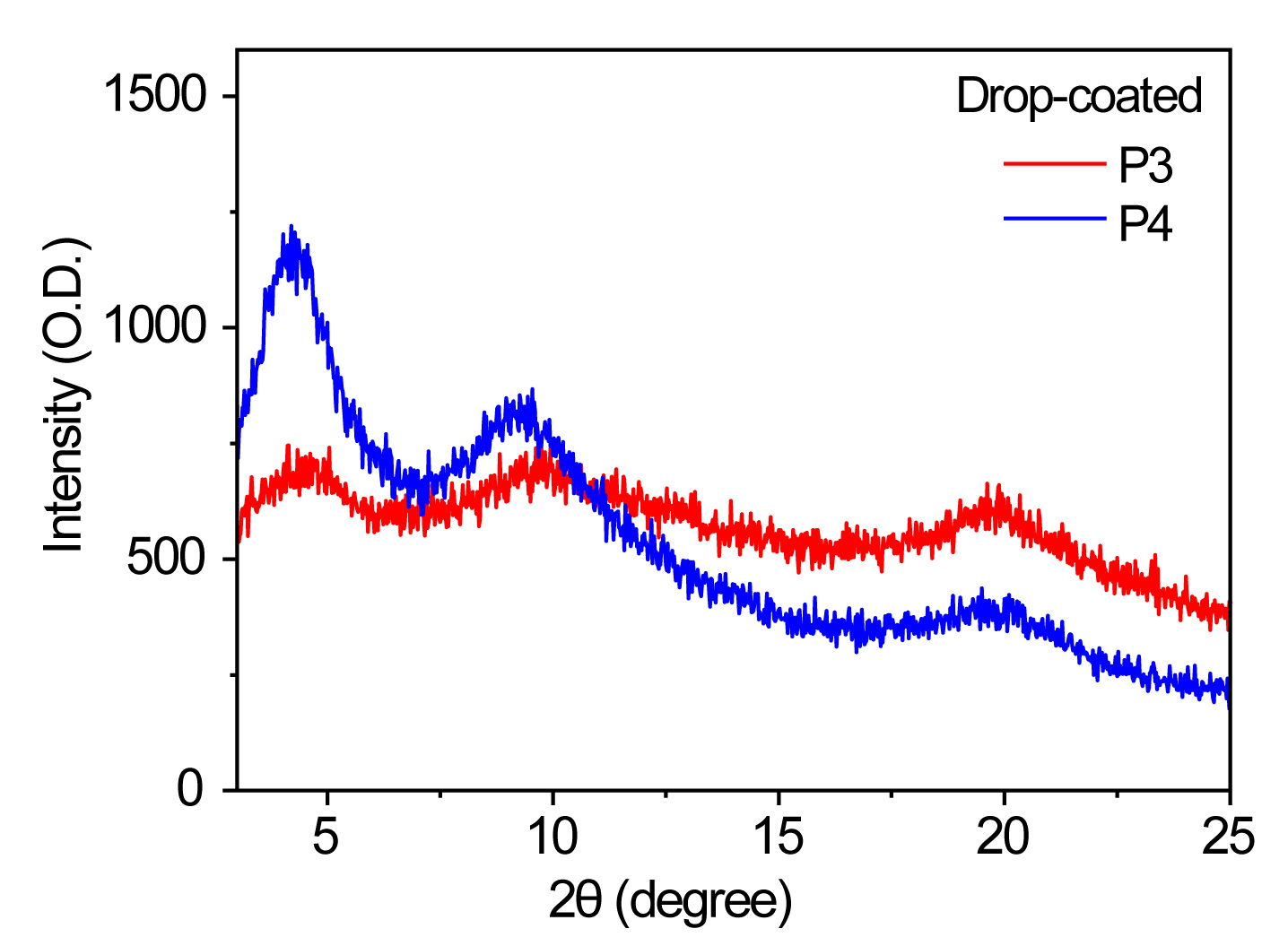
**

**Figure S13.** XRD curves of P3 and P4. The tested films were drop-coated from the gel solution at a concentration of 10 mg/mL.


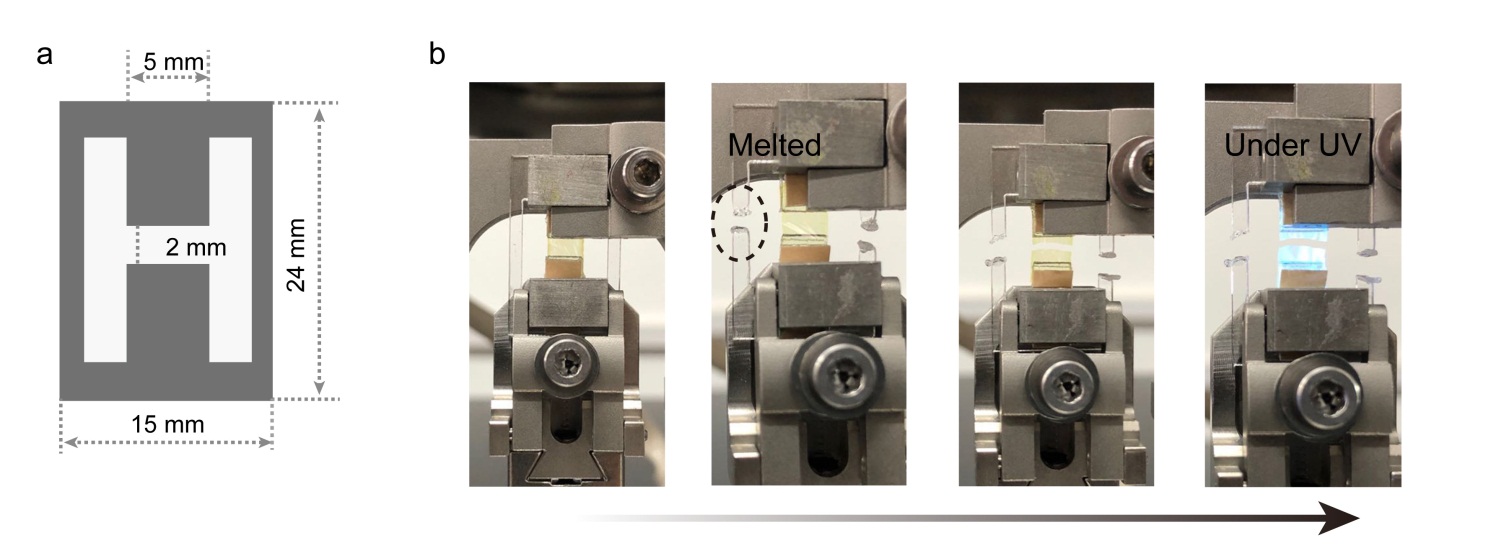


**Figure S14.** (a) The detailed size information of the hollow substrate. (b) Tensile strain process of the sample.


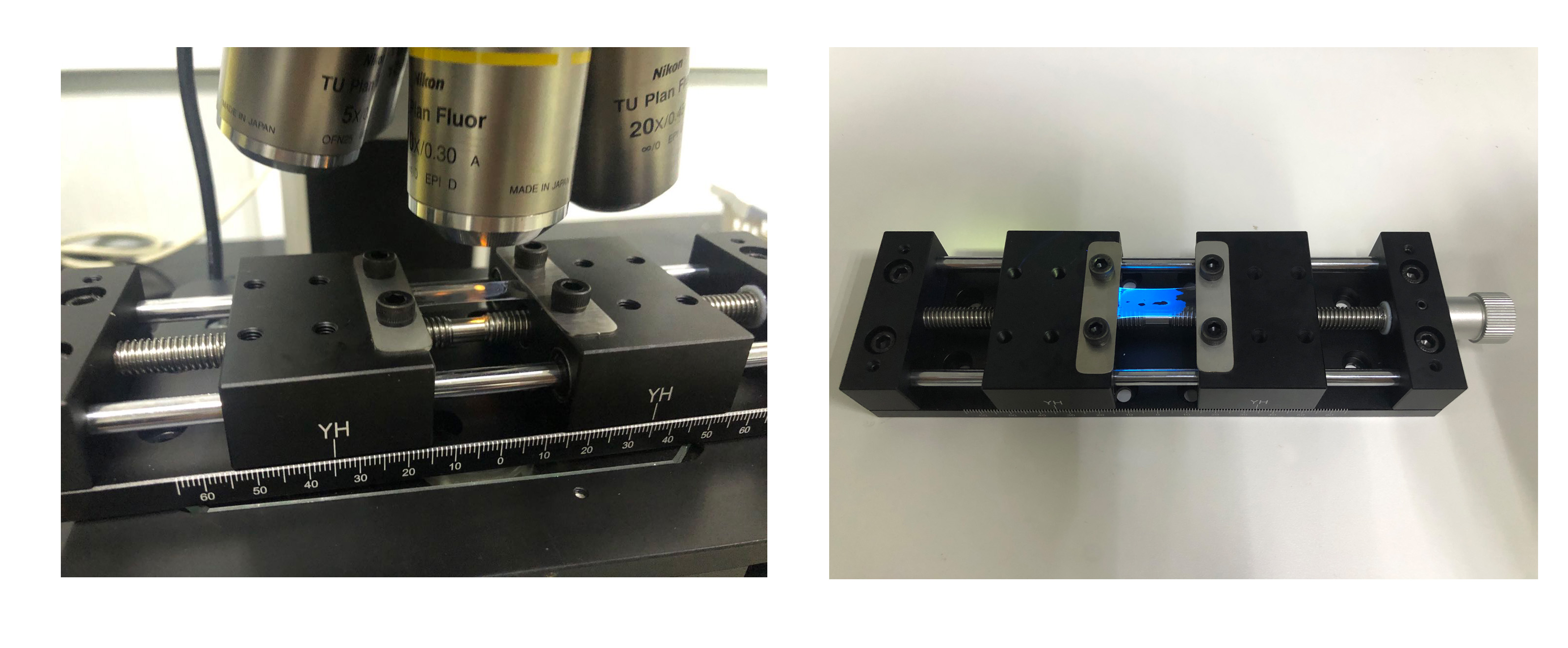


**Figure S15**. Photos of tensile the film under optical microscope with the FOE method.


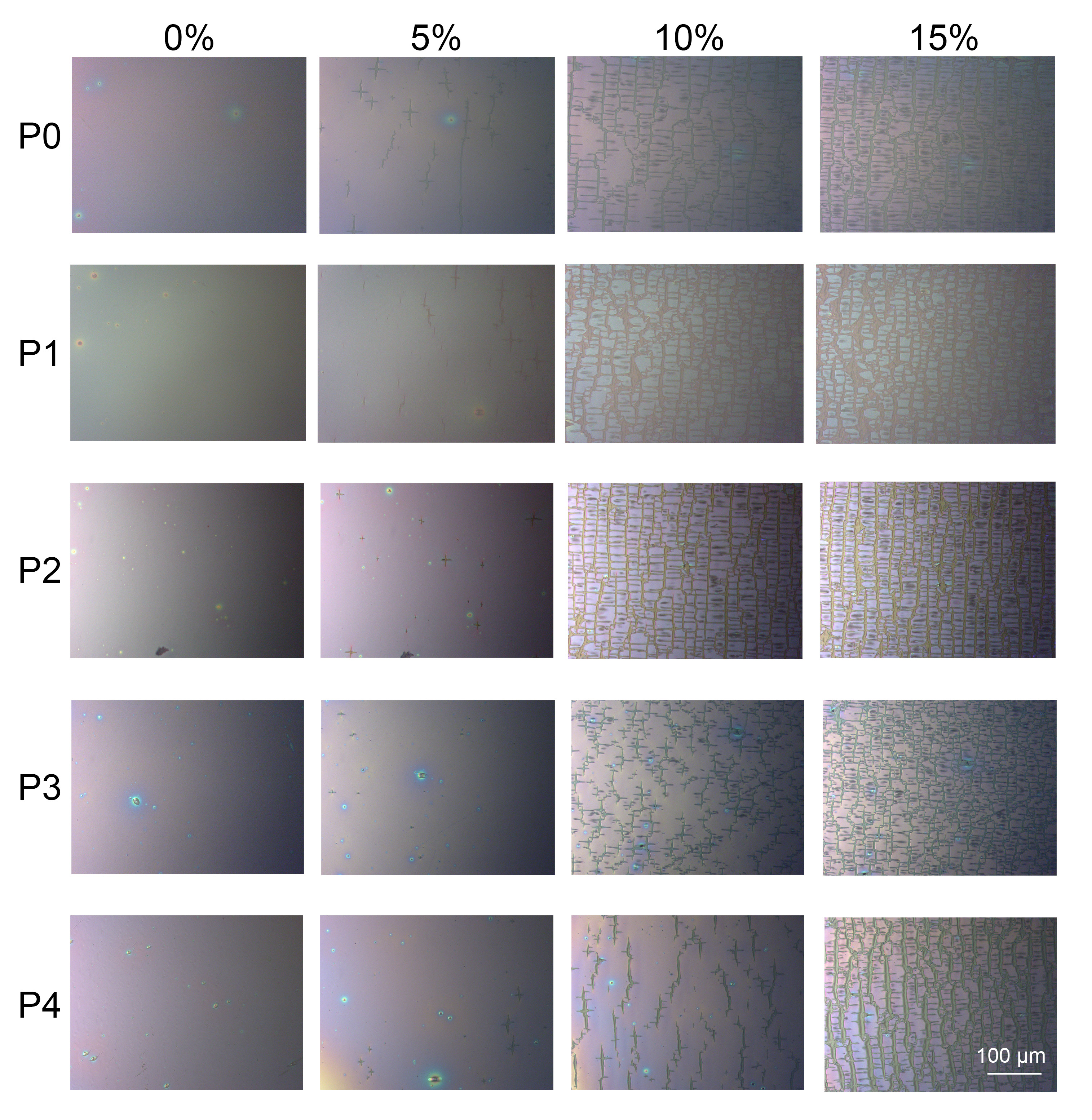


**Figure S16**. Optical microscope photos of the conjugated polymer films under strain from 0 ~15%, P0 is the referred materials (PODPF) without amide containing side chains.


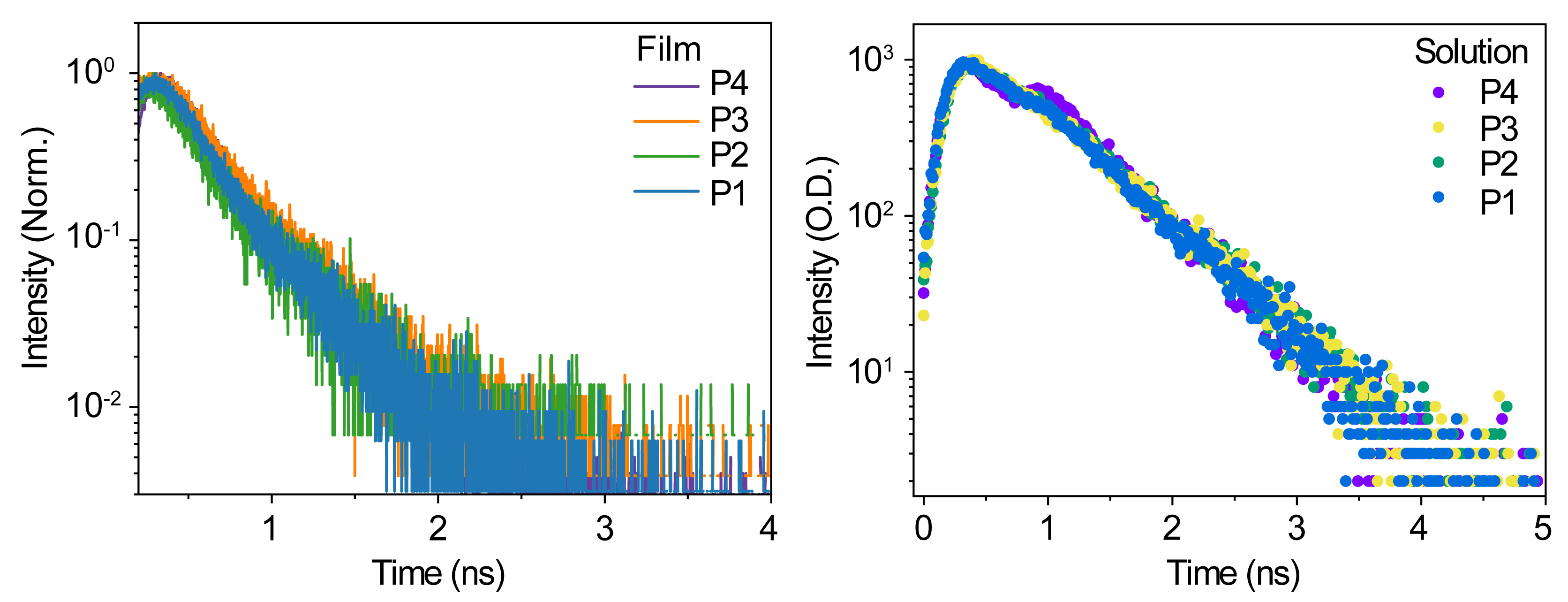


**Figure S17.** TCSPC measurment for the spin coated films, collected at the peak wavelength of 440 nm for the films and 430 nm for the solution.


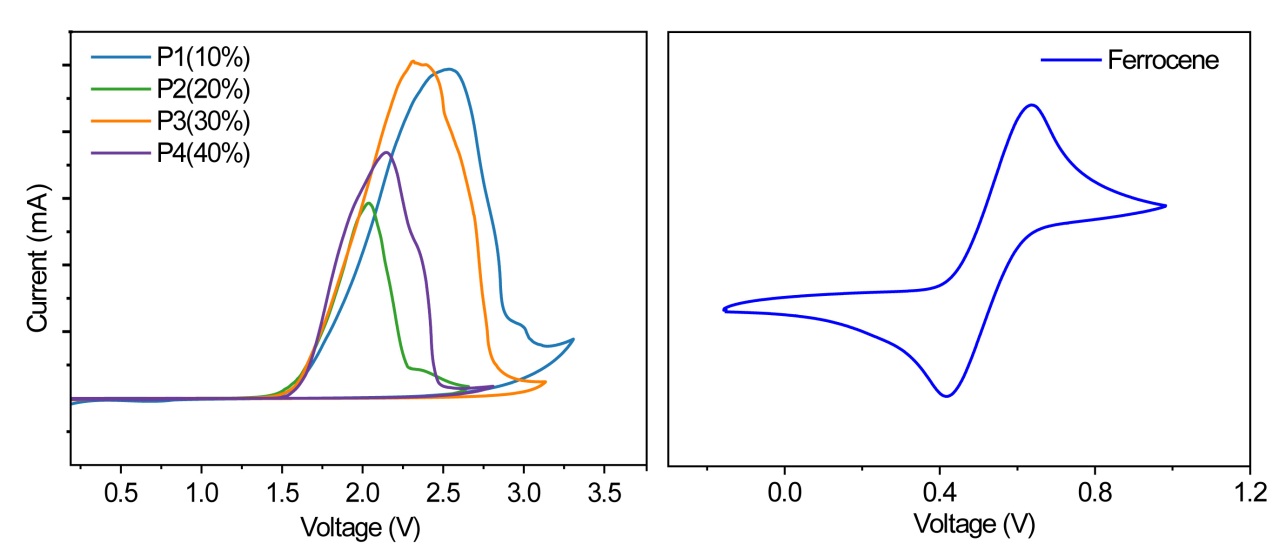


**Figure S18.** (left) Cyclic voltammograms curves (oxidation) of these copolymers; (right) Cyclic voltammograms curves of ferrocene.


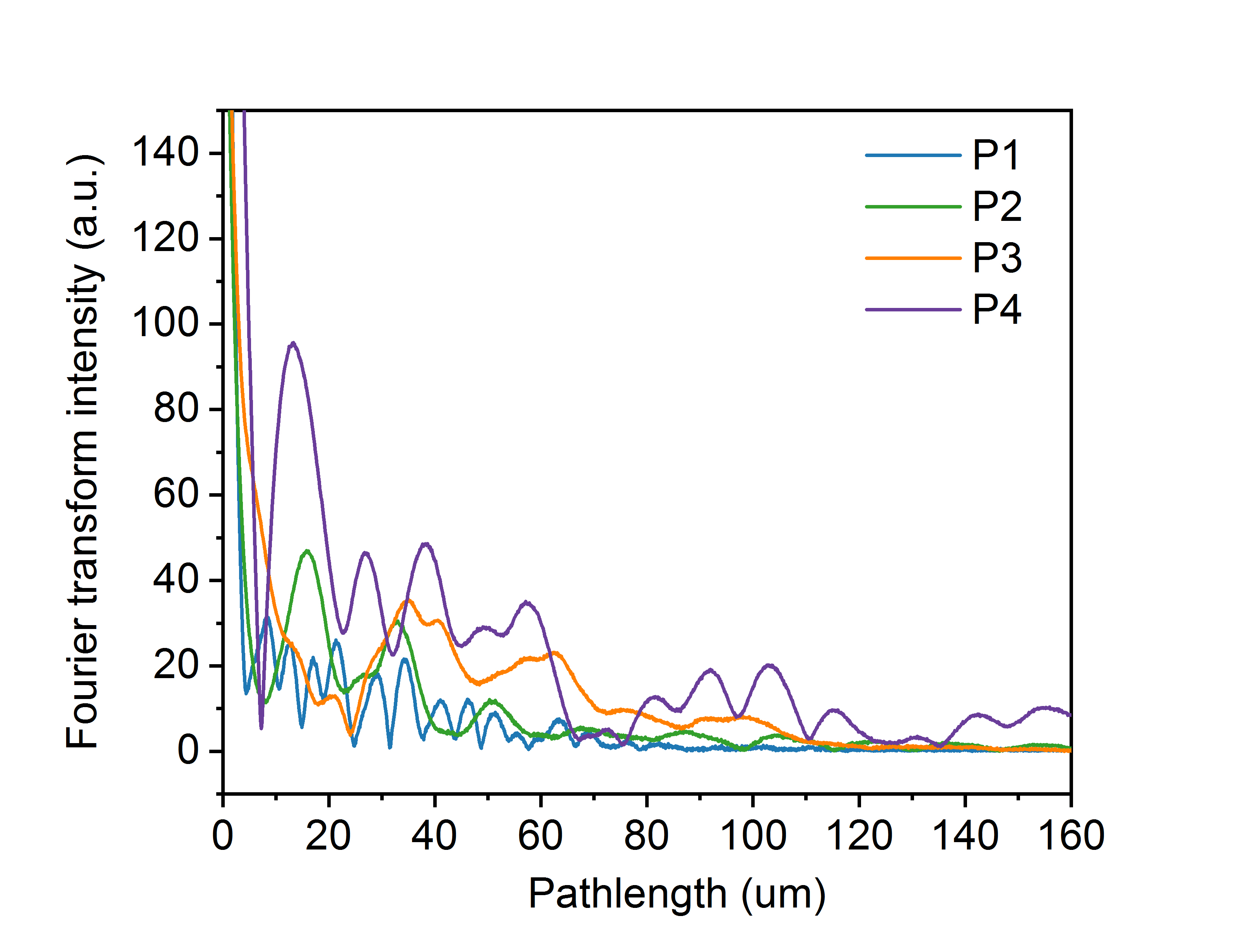


**Figure S19** The Fourier transform spectra of random laser emission spectra, showing the existence of a dominant cavity and its harmonics. The resonator loop (*L*) is calculated through the relation: *L* = 2dπ/n, where d is the FT length at which a peak is apparent in the transformed spectrum, n is the refractive index of these conjugated polymers (1.55).

**References**

[1] Rodriquez, D. et al., Comparison of Methods for Determining the Mechanical Properties of Semiconducting Polymer Films for Stretchable Electronics. *ACS Appl. Mater. Interfaces* **9**, 8855-8862 (2017).

[2] Song, R. et al., Unveiling the Stress–Strain Behavior of Conjugated Polymer Thin Films for Stretchable Device Applications. *Macromolecules* **53**, 1988-1997 (2020).

[3] Kim, J.-S. et al., Tuning Mechanical and Optoelectrical Properties of Poly(3-Hexylthiophene) through Systematic Regioregularity Control. *Macromolecules* **48**, 4339-4346 (2015).

[4] Ocheje, M. U., et al., Influence of Amide-Containing Side Chains on the Mechanical Properties of Diketopyrrolopyrrole-Based Polymers. *Polymer Chemistry* **9**, 5531-5542 (2018).

[5] Zeng, K. et al., Study of Mechanical Properties of Light-Emitting Polymer Films by Nano-Indentation Technique. *Thin Solid Films* **477**, 111-118 (2005).

[6]. Lin, J. et al., A Rational Molecular Design of *β*-Phase Polydiarylfluorenes: Synthesis, Morphology, and Organic Lasers. *Macromolecules* **47**, 1001-1007 (2014).

[7]. Lin, J. et al., Ultrastable Supramolecular Self-Encapsulated Wide-Bandgap Conjugated Polymers for Large-Area and Flexible Electroluminescent Devices. *Adv. Mater.* **31**, 1804811 (2019).
